# Tau Modulates mRNA Transcription, Alternative Polyadenylation Profiles of hnRNPs, Chromatin Remodeling and Spliceosome Complexes

**DOI:** 10.3389/fnmol.2021.742790

**Published:** 2021-12-03

**Authors:** Mauro Montalbano, Elizabeth Jaworski, Stephanie Garcia, Anna Ellsworth, Salome McAllen, Andrew Routh, Rakez Kayed

**Affiliations:** ^1^Mitchell Center for Neurodegenerative Diseases, University of Texas Medical Branch, Galveston, TX, United States; ^2^Departments of Neurology, Neuroscience and Cell Biology, University of Texas Medical Branch, Galveston, TX, United States; ^3^Department of Biochemistry and Molecular Biology, University of Texas Medical Branch, Galveston, TX, United States; ^4^Sealy Center for Structural Biology and Molecular Biophysics, University of Texas Medical Branch, Galveston, TX, United States

**Keywords:** alternative polyadenylation, frontal temporal dementia, gene ontology, gene set enrichment analysis, tau, neurodegenaration

## Abstract

Tau protein is a known contributor in several neurodegenerative diseases, including Alzheimer’s disease (AD) and frontotemporal dementia (FTD). It is well-established that tau forms pathological aggregates and fibrils in these diseases. Tau has been observed within the nuclei of neurons, but there is a gap in understanding regarding the mechanism by which tau modulates transcription. We are interested in the P301L mutation of tau, which has been associated with FTD and increased tau aggregation. Our study utilized tau-inducible HEK (iHEK) cells to reveal that WT and P301L tau distinctively alter the transcription and alternative polyadenylation (APA) profiles of numerous nuclear precursors mRNAs, which then translate to form proteins involved in chromatin remodeling and splicing. We isolated total mRNA before and after over-expressing tau and then performed Poly(A)-ClickSeq (PAC-Seq) to characterize mRNA expression and APA profiles. We characterized changes in Gene Ontology (GO) pathways using EnrichR and Gene Set Enrichment Analysis (GSEA). We observed that P301L tau up-regulates genes associated with reactive oxygen species responsiveness as well as genes involved in dendrite, microtubule, and nuclear body/speckle formation. The number of genes regulated by WT tau is greater than the mutant form, which indicates that the P301L mutation causes loss-of-function at the transcriptional level. WT tau up-regulates genes contributing to cytoskeleton-dependent intracellular transport, microglial activation, microtubule and nuclear chromatin organization, formation of nuclear bodies and speckles. Interestingly, both WT and P301L tau commonly down-regulate genes responsible for ubiquitin-proteosome system. In addition, WT tau significantly down-regulates several genes implicated in chromatin remodeling and nucleosome organization. Although there are limitations inherent to the model systems used, this study will improve understanding regarding the nuclear impact of tau at the transcriptional and post-transcriptional level. This study also illustrates the potential impact of P301L tau on the human brain genome during early phases of pathogenesis.

## Introduction

Tau is a neuronal protein found both inside and outside of the nucleus that contributes to the pathology of neurodegenerative diseases such as frontotemporal dementia (FTD) and Alzheimer’s disease (AD) (Sultan et al., 2011). It is primarily described as a microtubule-associated protein (Violet et al., 2014). Nuclear tau has been found to “protect” DNA (Hua and He, 2003; Sultan et al., 2011; Violet et al., 2014) during reactive oxygen species (ROS)-induced heat stress. However, nuclear and cytosolic tau interact with RNA to form droplets (Zhang et al., 2017) and aggregates (Kampers et al., 1996). Tau has also been observed altering nuclear structure (Monroy-Ramírez et al., 2013; Montalbano et al., 2019) in the human nuclei of neuroblastoma (Loomis et al., 1990; Shea and Cressman, 1998) and in HEK-293 cells. More specifically, phosphorylation of nuclear tau negatively regulates its nuclear function in pluripotent neuronal cells and neuroblastoma cells (Ulrich et al., 2018). Previous studies have revealed that nuclear tau plays a role in the DNA damage response (DDR) through deadenylation, which triggers major mRNA decay pathways (Baquero et al., 2019; Farmer et al., 2020). Most recently, we found that oligomeric assemblies of tau containing RNA-binding proteins impair chromatin remodeling and nuclear lamina formation through associations with histones and chromatin components in the nuclear compartment (Montalbano et al., 2020).

Despite the well-established importance of tau in the cytoskeleton of neurons (Venkatramani and Panda, 2019), there is growing evidence that tau is notably involved in nucleolar transcription and cellular stress responses (Maina et al., 2018a,b). Recently, it was shown that mutations and/or the phosphorylation of tau results in the deformation of the neuronal nuclear membrane and can disrupt nucleocytoplasmic transport (Lester and Parker, 2018) in FTD (Montalbano et al., 2019; Paonessa et al., 2019) and AD (Eftekharzadeh et al., 2018; Tripathi et al., 2019). Related studies analyzed the direct impact in transcriptional activity due to tau and found that nuclear tau regulates the expression of VGluT1, a gene that controls glutamatergic synaptic transmission, and that tau displacement from microtubules (MTs) increases nuclear accumulation of tau (Siano et al., 2019). Furthermore, tau modifies histone acetylation and was shown to have a broad epigenomic impact in the aging and pathology of AD human brains (Klein et al., 2019). It has also been observed that tau interacts with neuronal pericentromeric DNA regions, particularly in association with HP1 and H3K9me3 (Mansuroglu et al., 2016), this observation spots tau protein as potential chromatin remodeling factor. Lastly, tau exhibits binding interactions with genic and intergenic DNA sequences of primary cultured neurons, especially in positions ± 5,000 bp away from the start site of transcription (Benhelli-Mokrani et al., 2018).

In eukaryotic cells, the maturation of 3′ ends in mRNA involves endonucleolytic cleavage of the nascent RNA followed by the synthesis of a poly(A) tail on the 3′ terminus of the cleaved product by a poly(A) polymerase (PAP) (Stewart, 2019). This reaction is called polyadenylation and is fundamentally linked to transcription termination. The sequences for the mRNA precursors and the proteins required for polyadenylation are well understood. It has been clearly elucidated that a single gene can give rise to many possible transcripts, each with different polyadenylation sites [poly(A)-sites, or PASs], and that differential usage of these sites can lead to the formation of mRNA isoforms. This phenomenon is called alternative polyadenylation (APA) (Gruber and Zavolan, 2019) and is a common event in eukaryotic cells. In fact, researchers have determined that 50% of mammalian mRNA-encoding genes express APA isoforms (Tian et al., 2005; Shepard et al., 2011). Considering this information, we used tau-inducible HEK (iHEK) cell lines to obtain and analyze transcriptomic and APA profiles in the presence of WT and P301L tau. To characterize transcriptional and post-transcriptional profiles modified by WT and P301L, we utilized Poly(A)-ClickSeq (PAC-Seq) to measure changes in the expression of the host mRNA transcript whilst simultaneously characterizing changes in the PAS usage or creation of mRNA isoforms. In addition, we employed Gene Set Enrichment Analysis (GSEA) and Gene Ontology (GO) to study the main gene domains modulated by tau.

## Materials and Methods

### Cell Culture and Tau Expression

In this study we used two different versions of tau-inducible HEK (iHEK) cells: iHEK overexpressing WT tau and iHEK overexpressing mutated P301L tau. They were maintained in Dulbecco’s modified eagle medium (DMEM) supplemented with 10% fetal bovine serum (FBS) at 37^°^C in 5% CO_2_. To induce WT and mutant tau overexpression, iHEK cells were treated with 1 μg/ml of Tetracycline (Tet) for 24 h in FBS-depleted DMEM (Gibco™ LS11965118, Thermo Fisher Scientific). iHEK cells not treated with Tet were named control (Ctr). After 24 h, two washes with medium were done to remove excess Tet. Immediately after the washes, the cells were stained and collected. Detachment of cells was completed with Trypsin (GibcoTM Trypsin-EDTA, 0.25% Phenol red, LS25200114 Thermo Fisher Scientific), and the cells warmed for 3 min in the incubator following the addition of Trypsin. The cells were then centrifuged at 1,000 rpm for 5 min. Lastly, cell pellets were harvested and used for protein fractionation, and mRNA extraction.

### RNA Extraction

Total mRNA was collected by using TRIzol extraction reagent according to established protocol (Rio et al., 2010). RNA samples for Real Time Analysis (RT-PCR) were quantified using a NanoDrop Spectrophotometer (NanoDrop Technologies), followed by analysis on an RNA Nano chip using the Agilent 2100 Bioanalyzer (Agilent Technologies). Only samples with high quality total RNA were used (RIN: 7.5–10.0) for the study. Synthesis of cDNA was performed with either 0.5 or 1 μg of total RNA in a 20 μl reaction using the reagents available within the Taqman Reverse Transcription Reagents Kit from Life Technologies (#N8080234). Q-PCR amplifications (performed in duplicate or triplicate) were done using 1 μl of cDNA in a total volume of 20 μl using the iTaq Universal SYBR Green Supermix (Bio-Rad #1725125). The final concentrations of the primers were 300 nM. Relative RT-QPCR assays are performed with either 18S RNA gene as a normalizer. Absolute RNA quantification analysis was performed using known amounts of a synthetic transcript created from the gene of interest.

### Library Preparation Protocol

Protocols for Poly(A)-ClickSeq (PAC-Seq) have been described in detail by Jaworski and Routh (2018) and Elrod et al. (2019). Approximately 1 μg of total cellular RNA per sample was used as a template in reverse-transcription reactions supplemented with 40 μM Azido-VTPs and primed using an oligo-dT primer containing a partial Illumina i7 indexing adaptor. Azido-terminated cDNA fragments were “click-ligated” to hexynyl-functionalized click-adaptors containing the Illumina i5 universal sequencing adaptor. Single-stranded cDNA libraries were indexed in a final PCR reaction for 15–18 PCR cycles. Final libraries were size extracted by gel-electrophoresis and submitted for sequencing using an Illumina NextSeq550 to prepare 1 × 150 SE reads. RNAseq datasets is uploaded to NCBI SRA, reference number: PRJNA744518.

### Poly(A)-ClickSeq

PAC-Seq data were analyzed using the Differential Poly-A Clustering (*DPAC*) program, which ran with default settings as previously described (Routh, 2019). *DPAC* trims and quality-filters raw FASTQ data and therefore requires each read to have at least 25 “As” at the 3′ end of the read. These reads are then trimmed using *cutadapt*. Trimmed reads are mapped to the reference human genome (hg19) using *HISAT2* (Kim et al., 2019). The 3′end of mapped reads are thus used to annotate poly(A)-sites and annotated based upon overlaps with gene annotations obtained from UCSC genome browser. Gene counts were extracted and *DESeq2* was used to calculated changes in gene expression as well as relative changes in expression in individual poly(A)-sites found within single genes. Differential gene expression was assigned when a gene had a fold change greater than ± 1.5-fold with a p-adj value less than 0.1. Alternative polyadenylation is assigned when a single gene has two or more clustered poly(A)-sites wherein at least one of these sites has a differential usage greater than a ± 1.5-fold, a p-adj value less than 0.1, and a change of the relative usage of a poly(A)-cluster within the gene of greater than 10%.

### Western Blotting and Cell Fractioning

Immunoblot (IB) analyses were performed with iHEK cell fraction samples as previously described (Montalbano et al., 2020). Approximately 10 μg of protein preparations were loaded onto precast NuPAGE 4–12% Bis-Tris gels (NP0335BOX, Invitrogen) for sodium dodecyl sulfate-polyacrylamide gel electrophoresis (SDS-PAGE) analyses. Gels were subsequently transferred onto nitrocellulose membranes and blocked overnight at 4°C with 10% non-fat dry milk. Membranes were then probed for 1 h at room temperature with Pan-Tau (Tau13, 1:10,000, MMS-520R Covance), (GAPDH, 1:1,000, ab9485 Abcam), Histone3 (1:1,000, ab201456 Abcam), RCC1 (1:100, Clone E-6 sc-55559 Santa Cruz Tech.), DNAJC2 (1:5,000, ab134572 Abcam), Histone 1.2 (1:500, ab4086 Abcam), HMGB1 (1:500, ab18256 Abcam), SMARCA5 (1:10,000, #PA5-78253, Invitrogen), SMARCC1 (0.4 μg/ml, #PA5-55058, Invitrogen), and β-Actin (1:5,000, #A1978, Sigma-Aldrich). Antibodies were diluted in 5% non-fat dry milk. Immunoreactivity was detected using a horseradish peroxidase (HRP)-conjugated anti-rabbit immunoglobulin G (IgG, 1:10,000, NA934 GE Healthcare). Tau13 and Tau5 immunoreactivity were detected using an anti-mouse IgG (1:10,000, NA931 GE Healthcare) diluted in 5% milk. ECL Plus (K-12045-D50, GE Healthcare) was used to visualize protein bands. LaminB1/Histone3 and GAPDH were used to normalize and quantify nuclear and cytoplasmic proteins, respectively. The compartment extraction was conducted with Qproteome Cell Compartment Kits (Qiagen, #37502); nuclear, cell membrane, and cytoplasmic proteins were isolated and preserved for IB analysis.

### Immunofluorescence of Fixed Cells and Fluorescence Microscopy

Cells on a 24-well coverslip were fixed with 0.5 ml of 4% PFA/PBS for 15 min. The cells were then washed 3 times in phosphate buffered saline (PBS), for 5 min for each wash. The cells were permeabilized in 0.5 ml PBS and 0.2% Triton X-100 in phosphate buffered saline containing 0.5% Tween (PBST) for 5 min. Blocking was done in 0.5 ml of 5% normal goat serum (NGS) in PBST for 1 h. Primary antibody was diluted in 5% NGS/PBST overnight at 4°C for incubation, and then washed 3 times in PBST, for 10 min each. Secondary antibody diluted in 5% NGS/PBST was incubated for 2 h at room temperature. All the secondary antibodies were purchased from Thermo Fisher Scientific and used at a 1:800 dilution for staining. After applying secondary antibodies, cells were incubated in DAPI (nuclei staining) diluted 1:10,000 in PBST (5 mg/ml stock solution) for 5 min after the first wash. The cells were then washed 2 times with PBST, and once with PBS (10 min each) prior to mounting coverslips. Coverslips were mounted on glass microscope slides using 8–10 μl of Prolong Gold Antifade mounting media with DAPI (Invitrogen, P36941) per coverslip. Slides were air-dried in fume hood or stored at 4°C until ready to be dried in the fume hood. The primary antibodies used in this study for immunocytochemistry (ICC) are as follows: Histone 1.2 (Abcam ab4086—1 μg/ml), Ki-67 (Abcam ab92742—1 μg/ml), SMARCC1 (Invitrogen PA5-55058—0.25 μg/ml, SMARCA5 (Invitrogen PA5-78253—1 μg/ml, MCM2 (Abcam ab108935—1/1,000), RCC1 (Santa Cruz, Inc. sc-55559—1:50), and Tau13 (Bio Legend MMS-520R—1/200). After three washes with PBS, cells were probed with mouse and rabbit-specific fluorescent-labeled secondary antibodies (1:200, Alexa Fluor 488 and 633, Life Technologies). Single frame images were collected using the Keyence BZ-X 710 Microscope. Images for quantification of area and integrated density were taken in nuclear target areas guided by the DAPI fluorescence. We then performed single extraction analysis using BZ-X Analyzer software (Keyence). We used 200 nuclei per target area and used the Nikon 20X objective for imaging and quantification analysis.

### Statistical Analysis

All *in vitro* experiments were performed in at least three biological replicates. All data are presented as means ± SD and were analyzed using GraphPad Prism Software 6.0. Statistical analyses included the Student’s *t*-test or one-way ANOVA followed by Tukey’s Multiple Comparisons Test. Column means were compared using one-way ANOVA with treatment as the independent variable. In addition, group means were compared using two-way ANOVA considering factors for each treatment, respectively. When ANOVA showed a significant difference, pair-wise comparisons between group means were examined by the Tukey and Dunnett Multiple Comparison Test.

## Results

### WT Tau Up-Regulates Genes Associated With Cytoskeleton Organization and Nuclear Speckles/Bodies

**FIGURE 1:**
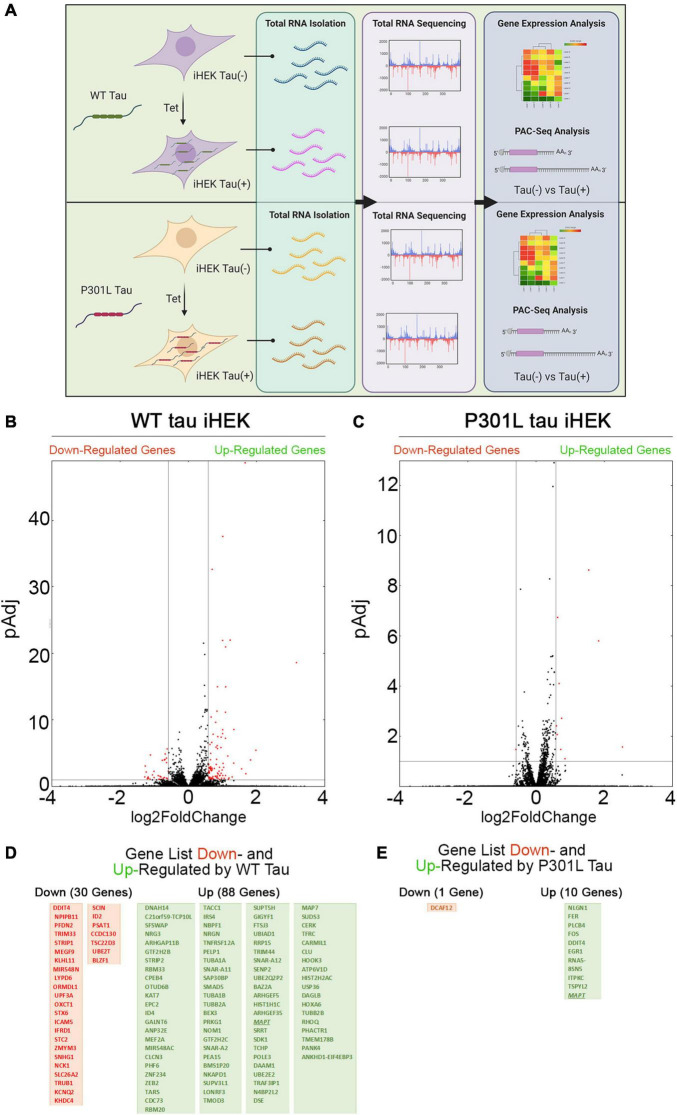
Tau-dependent Gene expression. **(A)** Schematic representation of experimental plan, from Tet induction in WT and P301L Tau iHEK to RNA isolation, sequencing to gene expression analysis. **(B)** Volcano Plot for Down- and Up-regulated gene in WT tau iHEK. **(C)** Volcano Plot for Down- and Up-regulated gene in P301L tau iHEK. **(D)** Gene Lists of Down-Regulated (Red Boxes) and Up-Regulated (Green Boxes) Genes in WT tau iHEK. **(E)** Gene Lists of Down-Regulated (Red Boxes) and Up-Regulated (Green Boxes) Genes in P301L tau iHEK.

Firstly, we evaluated changes in gene expression profiles upon expression of WT and P301L tau in iHEK cells that were induced with tetracycline (Tet). After 24 h of Tet induction, we confirmed tau expression in the cytoplasm and nuclei of iHEK cells (Supplementary Figure 1A). Total cellular RNA from WT and P301L tau [untreated (Control) and treated (+ Tet)] study groups was extracted using TRIzol reagent and by following established protocol (Montalbano et al., 2019, 2020). RNA was sequenced using Poly(A)-ClickSeq (PAC-Seq) to measure changes in gene expression and poly(A)-site usage (Elrod et al., 2019). A schematic of the experimental design is provided in Figure 1A. Volcano scatterplots from WT and P301L tau iHEK (Figures 1B,C, respectively) demonstrate a substantial difference in the number of genes regulated by WT tau and P301L tau. After Tet induction in the WT tau iHEK cell system, we observed up-regulation of 88 genes and down-regulation of 30 genes (gene names listed in Figure 1D). In the P301L tau iHEK cell system, these numbers dropped to 10 up-regulated genes and only 1 down-regulated (gene names listed in Figure 1E).

**FIGURE 2:**
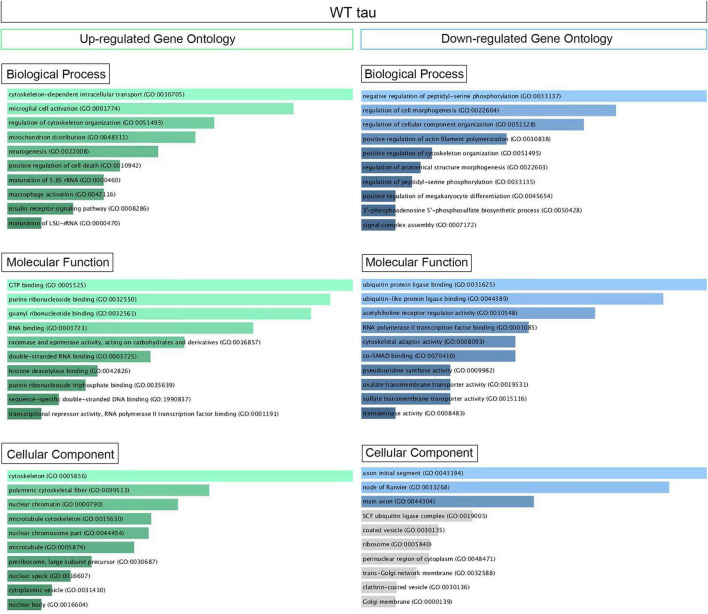
Up- and Down regulated genes in WT Tau iHEK Gene Ontology. Left Column (Green) Up-regulated genes analyzed by Enrich GO and divided by Biological Process, Molecular Function and Cellular Component. Right Column (Blue) Down-regulated genes analyzed by Enrich GO and divided by Biological Process, Molecular Function and Cellular Component. Gray bars represent not significant correlation.

Supplementary Figure 1B displays the scatterplots of WT and P301L tau gene expression, while Supplementary Figure 1C reports the Principal Component Analysis (PCA). PCA demonstrates significant variation among the study groups. More specifically, the analysis suggests a significant difference in transcriptional activity of WT tau due to the higher number of genes modulated in comparison to the mutant P301L tau form. Using EnrichR (Kuleshov et al., 2016), we established Gene Ontology (GO) of the biological processes, molecular functions, and cellular components altered by both the up-regulated and the down-regulated sets of genes. WT tau GO is summarized in Figure 2. WT tau up-regulated genes belonging mainly to classes of cytoskeleton-dependent intracellular transport genes (GO: 0030705, TUBA1A, TUBB2B TUBA1B, TUBB2A, and HOOK3) and genes responsible for the regulation of cytoskeleton organization (GO: 0051493). Imbalanced expression of tubulin and tau induces neuronal dysfunction in *C. elegans* (Miyasaka et al., 2018), indicating that tau itself can disturb tubulin gene expression. The reason behind this pronounced involvement of TUBB genes could be due to the fact that *TUBB1B, TUBB2B, TUBA1A*, and *TUBB2A* are clustered together within the genome (Bittermann et al., 2019).

It is important to note that biological process such as microglial cell activation (GO: 0001774) and macrophage activation (GO: 0042116) were also observed as being up-regulated, which confirms known effects of tau on the neuro-inflammatory response commonly observed in neurodegenerative diseases (Kwon and Koh, 2020). Neuro-immunomodulation can also effect cytoskeleton reorganization (Morales et al., 2013). Our GO analysis revealed up-regulated genes involved in mitochondrion distribution (GO: 0048311, *MAPT*, and *MEF2A* genes), morphogenesis (GO: 0070584, *SUPV3L1, p* = 0.03892), neurogenesis (GO 0022008, *NOM1, MAPT*, and *DAGLB* genes), and positive regulation of cell death (GO 0010942, *SAP30BP, MAPT*, and *CLU* genes). The increase of *CLU* expression was a particularly interesting observation. Clusterin is a multifunctional, secreted chaperone involved in several basic biological events, including cell death, tumor progression and neurodegeneration. The *CLU* gene is notably associated with an increased AD risk (Karch and Goate, 2015). In terms of molecular functions, the up-regulated genes we observed have several enriched pathways, including RNA binding (GO: 0003723, *USP36, NOM1, TFRC, BAZ2A, SUPTSH, PHF6, FTSJ3, SUPV3L1, TUBA1B, RBM20, MAPT, RBM33, PELP1, HIST1H1C, CPEB4*) and several nuclear functions, such as histone deacetylase binding (GO: 0042826, *MEF2A, SUDS3* and *PHF6*) and sequence-specific double stranded DNA binding (GO: 1990837, *MEF2A, KAT7*, and *MAPT*).

Transcriptional products of up-regulated genes are mostly localized in the cytoplasm and nuclear compartments. We detected transcripts associated with nuclear chromatin (GO: 0000790), such as *MEF2A, ZEB2, ANP32E, SUDS3, HIST2H2AC*, and *HIST1H1C*. We also examined nuclear speck transcripts (GO: 0016607), such as *CARMIL1, USP36, GTF2H2C, BAZ2A*, and *MAPT* genes, which are also included in nuclear body components (GO: 0016604), along with *SUDS3* and *SENP2*. The other cell compartment well represented in our GO analysis is the cytoplasm. In particular, the microtubule cytoskeleton (GO: 0015630) contained the following up-regulated genes: *TUBB2B, SAP30BP, TUBA1B, TUBA1A, TMOD3, MAP7, TARS, TACC1, MAPT, CLU*, and *RHOQ*. A complete Enrich-GO list of significant up-regulated genes observed in WT tau is presented in Supplementary Table 1.

### WT Tau Down-Regulates Genes Involved in Ubiquitin-Related Processes as Well as Genes Associated With Golgi and Mitochondrial Components

Overall, thirty genes were significantly downregulated by WT tau protein. The main biological process affected was the regulation of cellular component organization (GO: 0051128) as it relates to cytoskeleton organization and structure morphogenesis. Molecular functions associated with the aforementioned genes are closely related to ubiquitin protein ligase binding (GO: 0031625) and ubiquitin-like protein ligase binding (GO: 0044389). Genes important to neuronal components included genes essential to the structure of initial axonal segments, nodes of Ranvier, and main axons. These three groups typically involve the gene *KCNQ2*. This gene encodes for Potassium voltage-gated channel subfamily KQT member 2, which plays a critical role in determining the subthreshold electrical excitability of neurons as well as the responsiveness of neurons to synaptic inputs. Therefore, KCNQ2 is important in the regulation of neuronal excitability and the loss-of-function or gain-of function of this gene can lead to various forms of neonatal epilepsy (Niday et al., 2017).

Furthermore, Cullin-RING E3 ubiquitin-ligase complex component *KLHL11* is down-regulated, as well as the *STX6* gene. STX6 encodes for Syntaxin-6, which is involved in intracellular vesicle trafficking and is integrally associated with the Golgi apparatus. Another Golgi protein that is down-regulated is Golgin-45 (*BLZF1*). It is required for normal Golgi structure and for protein transport from the Endoplasmic Reticulum (ER) through the Golgi apparatus to the cell surface (Short et al., 2001). Lastly, the ER gene *STC2* is downregulated and encodes for Stanniocalcin-2. This glycoprotein has an anti-hypocalcemic action on calcium and phosphate homeostasis (Ito et al., 2004).

We also detected two nucleolus-localized genes among the down-regulated group: UBE2T (ubiquitin-conjugating enzyme with E2 T) and UPF3A, (a regulator of non-sense transcript 3A). The mitochondrial genes that were down-regulated included OXCT1 (Succinyl-CoA: 3-ketoacid coenzyme A transferase 1, mitochondrial enzyme), TRUB1 and PFDN2 (Prefoldin subunit 2). An Enrich-GO list of downregulated genes present in WT tau is depicted in Supplementary Table 2.

Although there are limitations inherent with the model used, these data suggest that WT tau intrinsically and significantly impacts the cell at a transcriptional level. More specifically, a higher number of genes are up-regulated and down-regulated by WT tau when compared to P301L tau. This suggests that the P301L mutation of tau has not impact at the transcriptional level. This sort of loss could have detrimental effects on cell structure and organization.

### P301L Tau Up-Regulates Gene Expression of Components Related to Axonal Microtubule Skeleton, Nuclear Speckles, and Ribonucleoprotein

The GO pathways and cellular compartments upregulated and downregulated by P301L tau are listed In Supplementary Tables 3, 4, respectively. As observed in WT tau iHEK cells, the *MAPT* gene is on the upregulated gene list for P301L tau, as expected after Tet induction of the iHEK cells. Within the group of axonal and cytoskeleton genes, we noticed up-regulation of NLGN1, a gene that encodes for Neuroligin-1. Neuroligin is a postsynaptic neuronal surface protein involved in cell-to-cell interactions via its interactions with neurexin family members (Bemben et al., 2015). It has been established that the NLGN1 gene is associated with amyloid-β oligomers (AβOs) in AD-causing synaptic impairment (Brito-Moreira et al., 2017). In addition, NLGN1 is typically altered in AD hippocampi and also modulates amyloid-beta oligomer toxicity (Dufort-Gervais et al., 2020). Neuroligin-1 plays an influential role in synaptic function and synaptic signal transmission, most likely through its ability to recruit and cluster together other synaptic proteins (Bemben et al., 2015). For instance, neuroligin-1 may promote the initial formation of synapses (Craig and Kang, 2007), but is not essential for the complete formation of synapsyes. *In vitro*, Neuroligin-1 triggers the *de novo* formation of presynaptic structures. NLGN1 may also be involved in specification of excitatory synapses (Bemben et al., 2015). For example, NLGN1 functions to maintain wakefulness quality and normal synchrony of cerebral cortex activity during wakefulness and sleep (El Helou et al., 2013). Neuroligin-1 is predominantly located in synaptic cleff of the cell membrane (Wu et al., 2019).

When we analyzed upregulated genes, we detected a considerable number of genes related to nuclear body (GO: 0016604) and nuclear speck (GO: 0016607) domains including the genes *ITPKC* and *MAPT*. Interestingly, it has been observed that the *FER* gene participates in several different cytoplasmic and nuclear functions. For example, *FER* is associated with nuclear chromatin (GO: 0000790) and the microtubule skeleton (GO: 0015630). The *FER* gene also encodes for a tyrosine-protein kinase that plays a role in synapse organization, trafficking of synaptic vesicles, the generation of excitatory post-synaptic currents, and neuron-to-neuron synaptic transmission (Lee et al., 2008). Lastly, FER plays a role in neuronal cell death after brain damage (Lee et al., 2008). The only gene down-regulated by P301L tau is *DCAF12*, which is a component of the Cullin-RING ubiquitin ligase complex (Patrón et al., 2019). This gene is also down-regulated by WT tau and belongs to genes associated with ubiquitinization processes. The failure of ubiquitinization pathways is known to have a strong connection to neurodegenrative diseases (Zheng et al., 2014). Supplementary Figure 2 summerizes upregulated and downregulated genes in P301L tau, subcatagorized by biological process, molecular function and cellular component.

In summary, the P301L mutation upregulates genes involved in positive regulation of neuronal death and responsiveness to reactive oxygen species (ROS) production. This is in contrast to the genes altered by WT tau that have a greater effect on cell structural processes. The most important molecular function altered by such genes would be sequence-specific double-stranded DNA binding, transcriptional expression, and chromatin remodeling. Overall, our GO data suggests no impact on transcription in mutated P301L tau that may relate to pathology only through the protein level resulting in toxic aggregation. Modulating genes known to be associated with neurodegenerative disease suggests that mutated tau engenders harmful transcription patterns that contribute to the well-established effects of tau proteinaceous-aggregation toxicity.

### WT Tau Modulates Gene Expression of Chromatin Organization and Remodeling Factors

**FIGURE 3:**
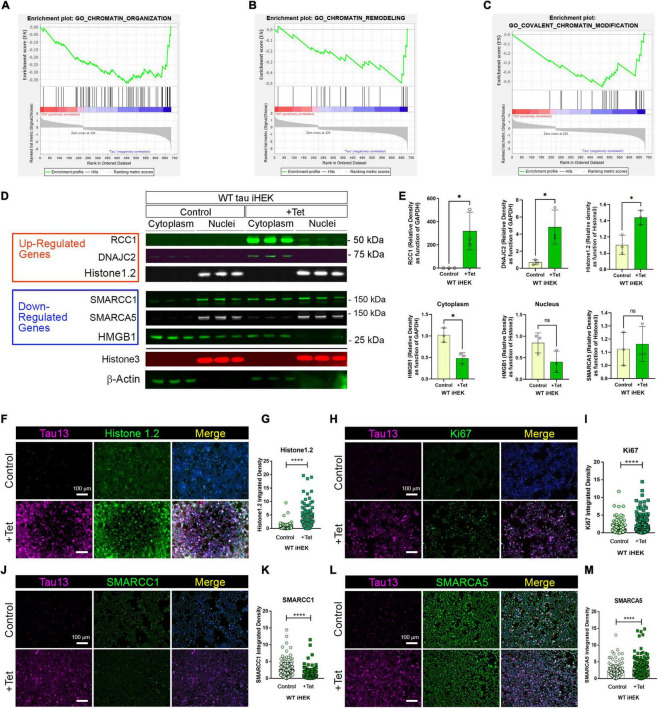
WT tau modulates gene expression of chromatin organization and remodeling factors. **(A)** Enrichment plot for GO Chromatin organization. **(B)** Enrichment plot for GO Chromatin remodeling. **(C)** Enrichment plot for GO-Covalent Chromatin modification. **(D)** IB of Up-regulated genes: RCC1, DNAJC2 and Histone 1.2 (red box) and Down-regulated genes: SMARCC1, SMARCA5 and HMGB1 (blue box) in cytoplasm and nuclear fractions from WT and P301L tau iHEK. Histone 3 and β-Actin has been used as loading control for nuclear and cytoplasmatic fractions, respectively. **(E)** Immunoblot quantification of RCC1 (*p < 0.01), DNAJC2 (*p < 0.01), Histone1.2 (*p < 0.01), cytoplasmic HMGB1 (*p < 0.01), nuclear HMGB1 and SMARCA5 proteins in cytoplasmatic and nuclear fractions of WT tau iHEK. **(F)** Representative Tau 13 (magenta) and Histone 1.2 (green) Co-IF of control (-Tet) and treated WT tau iHEK. **(G)** Histone 1.2 integrated density quantification in control and + Tet cells (Unpaired *t*-test, ****p < 0.0001). **(H)** Representative Tau 13 (magenta) and Ki67 (green) Co-IF of control (-Tet) and treated WT tau iHEK. **(I)** Ki67 integrated density quantification in control and + Tet cells (Unpaired *t*-test, ****p < 0.0001). **(J)** Representative Tau 13 (magenta) and SMARCC1 (green) Co-IF of control (-Tet) and treated WT tau iHEK. **(K)** SMARCC1 integrated density quantification in control and + Tet cells (Unpaired *t*-test, ****p < 0.0001). **(L)** Representative Tau 13 (magenta) and SMARCA5 (green) Co-IF of control (-Tet) and treated WT tau iHEK. **(M)** SMARCA5 integrated density quantification in control and + Tet cells (Unpaired *t*-test, ****p < 0.0001).

Gene Set Enrichment Analysis (GSEA) offers an opportunity to evaluate and identify classes of genes or proteins that are over-represented in a large set of genes or proteins and may have an association with disease phenotypes. Due to the differences in gene numbers modulated by WT tau vs. P301L tau, we performed GSEA. This analysis compared models with and without WT tau. We observed that WT tau down-regulates the expression of numerous genes linked to chromatin organization (Figure 3A) and chromatin remodeling (Figure 3B) domains. By looking at the chromatin organization and remodeling gene clusters, we identified that several high-mobility group box proteins (HMG) *HMGN5, HMGB2*, and *HMGA1* are up-regulated while *HMGB1* and *HMGN1* are down-regulated. It is important to note that HMGB1 is an activator of neuro-inflammatory responses and has been implicated in AD (Paudel et al., 2020). In addition, several components of the SWI/SFN chromatin remodeling complex are downregulated. The identification of genes *SMARCE1*, *SMARCA5*, and *SMARCC1*, imply that tau has a substantial impact on chromatin remodeling in the cells. The heterogeneous nuclear ribonucleoproteins (hnRNPs), *HNRNPU* and *HNRNPC*, were also found to be downregulated in WT tau. Down-regulation of several factors implicated in DNA replication and repair processes, indicates that WT tau also significantly affects the nuclear compartment of cells in terms of structure and content. Several of these genes are clustered as covalent chromatin modification in GO (Figure 3C).

To validate gene expression changes observed in GSEA analysis, we verified multiple proteins via western blot by using the up-regulated and down-regulated lists generated from Histone Binding GO. We verified up-regulation of RCC1, DnaJC2 and Histone1.2 proteins in the cytoplasm and in nuclear fractions of WT tau iHEK cells (Figure 3D). We also confirmed RCC1 expression and noticed its accumulation in the cytoplasm for both cell lines. Interestingly, we discerned that RCC1 is not imported into the nuclei where it should function as a regulator of chromatin condensation. Instead, DnaJC2 in P301L tau iHEK cells appear to be downregulated (Supplementary Figure 3). However, Histone 1.2 is upregulated in both cell lines. We did not observe down-regulation of the chromatin remodeling complex factors SMARCC1 and SMARCA5. Instead, we detected their accumulation in the cytoplasmic fractions while in the presence of tau, which suggests a deficit in these factors in the nuclei, as observed in our western blots. Lastly, HMGB1 and β-Actin are down-regulated, but HMGB1 is not detected in the nuclei when in the presence of tau. Histone 3 was used as a nuclear loading control. We performed quantification of bands to compare protein levels in WT tau iHEK of RCC1, DNAJC2, Histone1.2, HMGB1 (Cytoplasm/Nuclei) and SMARCA5 (Figure 3E).

To verify gene expression results, alongside western blots, we performed co-immunofluorescence in WT tau iHEK cells. We evaluated integrated density of Histone 1.2 (Figures 3F,G), Ki67 (Figures 3H,I), SMARCC1 (Figures 3J,K), and SMARCA5 (Figures 3L,M). Analysis was performed by considering nuclear integrated density of “–” and “+” tau WT iHEK proteins. To detect and confirm tau expression, we used the Tau13 antibody. MCM2 and RCC1 images and their relative integrated density quantifications are presented in Supplementary Figure 4.

**FIGURE 4:**
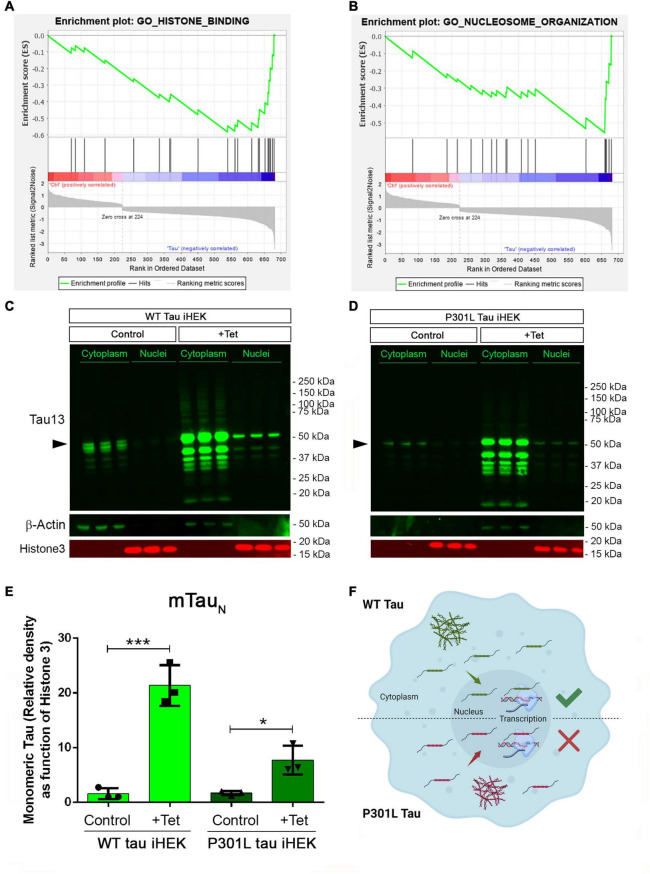
Tau nuclear shuttling. **(A)** GSEA GO-Histone Binding heat map in WT tau. **(B)** GSEA GO-Nucleosome organization heat map in WT tau. **(C)** Immunoblot (IB) with Tau13 (1:1,000) and β-Actin (1:1,000) of cytoplasm and nuclear fraction from WT (left panel) and **(D)** P301L (right panel) tau induced with Tet. Monomeric tau is indicated by rightwards filled arrows **(E)** Relative density of nuclear monomeric tau (mTauN, normalized with Histone3). Unpaired *t-test* has been performed to compare column means [(–) WT tau vs. WT tau ***p = 0.0009, (–) P301l tau vs. P301L tau *p = 0.0169]. **(F)** Schematic model on tau nuclear import in the two iHEK cell lines.

GSEA analysis for WT tau revealed significant down-regulation in the pathways for histone-binding (Figure 4A) and nucleosome organization clusters (Figure 4B). Several genes were detected in the histone and nucleosome domains, which were recurring and can be viewed in the chromatin gene list showed in Figure 3. In addition, we observed an up-regulation of *RCC1* (a regulator of chromosome condensation), *CTSL* (Cathepsin L), *MCM2* (Minichromosome maintenance complex component 2), and *DNAJC2* (DnaJ heat shock protein member C2). In Nucleosome GO, we observed up-regulated *HMGB2* and *HMGA1* (high mobility group box B2 and A1). On the contrary, several Lysine acetylation regulators were downregulated: *BRD3* and *BRD9* (from BRD family), *HDAC2, KDM5B*, *KAT7, and SFTD2*.

We also used western blotting to verify tau levels in cytoplasm and nuclear fractions of WT and P301L tau iHEK cells (Figures 4C,D, respectively). We found that upon Tet induction in both compartments, tau was detected, which was previously observed (Montalbano et al., 2019) and expected. Western blot analysis demonstrated that tau is represented mainly in its monomeric form (mTauN) when probing the nucleus. We compared the level of mTauN in both cell lines and we determined that mTauN increased in both cell lines after Tet induction. However, the WT mTauN was present in a significantly higher level when compared to the P301L mTauN (Figure 4E). This difference is due to the higher *MAPT* transgene expression efficiency in WT tau iHEK cell lines as was confirmed by RT-qPCR in a previous study (Montalbano et al., 2019). These observations suggest that the monomeric form of tau protein predominantly carries out transcriptional activity and that the P301L mutation did not affect the nuclear import of tau, but instead modulated transcriptional activity. Cytoplasmic mTau was quantified as well (Supplementary Figure 4). In general, we propose that WT and P301L tau both shuttle into the nuclei but then modulate transcription differently. The schematic model for this idea is represented in Figure 4F. In summary, many nuclear factor genes involved in several nuclear activities, including chromatin condensation, are downregulated in WT tau, which indicates a potential role of WT and P301L tau in the control of chromatin factors, expression and subsequent cellular localization.

### RNA Metabolism, Chromatin Organization and *HNRNP*s Precursor’s Display Shortened APAs in the Presence of WT Tau

**FIGURE 5:**
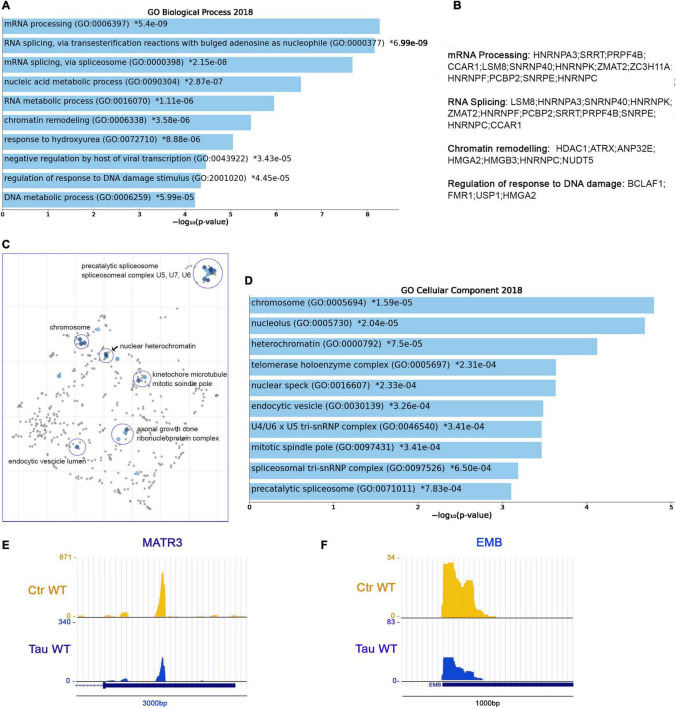
RNA metabolism, chromatin organization and *HNRNP*s precursor’s display shortened APAs in the presence of WT tau. **(A)** Enrich-GO Biological Process of WT tau shortened APAs (*p*-value reported). **(B)** Partial list of biological process genes with shortened APAs upon presence of WT tau (mRNA processing, RNA splicing, chromatin remodeling and regulation of response to DNA damage). **(C)** The Cellular Components scatterplot is organized so that similar gene sets are clustered together. The larger blue points represent significantly enriched terms—the darker the blue, the more significant the term and the smaller the *p*-value. The gray points are not significant. Plots has been generated and downloaded using scatter plot visualization Appyter. **(D)** Enrich-GO Cellular Component of WT tau shortened APAs (*p*-value reported). Representative poly-A site usage for shortened poly-A tails from PAC-Seq of MATR3 **(E)** and EMB **(F)** transcripts. An asterisk (*) next to a *p*-value indicates the term also has a significant adjusted *p*-value (<0.05).

From PAC-seq analysis, we identified 110 genes with shortened 3′UTRs. The majority of these shortened genes belong to significant pathways associated with mRNA processing (GO: 0006397), RNA Splicing (GO: 0000377, GO: 0000398), and RNA metabolic processes (GO: 0016070) (Figure 5A). These domains share several genes: *HNRNPA3, SRRT, PRPF4B, CCAR1, LSM8; SNRNP40, HNRNPK, ZMAT2, ZC3H11A, HNRNPF, PCBP2, SNRPE*, and *HNRNPC*. The regulation of responses to DNA damage (GO: 2001020) comprise the following genes: *BCLAF1, FMR1, USP1*, and *HMGA2* (others listed in Figure 5B).

Within the shortened APA precursors, various genes are related to nuclear function, such as the chromosome related genes (GO: 0005694) *IK, FMR1, HMGA2, SMC4, SMC3, SMC2*, and *SMC6*. Structural maintenance of chromosome (SMC) proteins are ATPases that are essential to chromosomal condensation, sister-chromatid cohesion, recombination, DNA repair, and epigenetic silencing of gene expression (Yatskevich et al., 2019). Eukaryotes have at least six genes encoding SMCs (SMC1-SMC6) (Aragon et al., 2013). They inherently work as heterodimers: SMC1/SMC3 (Cohesion Complex), SMC2/SMC4 (Condensin Complex) and SMC5/SMC6 (Aragon et al., 2013).

Several nucleolar (GO: 0005730) genes have altered poly(A) site usage by WT tau including: *PARP1, FMR1, CHD7, DDX21, PWP1, PPM1E, SMC2, RSL1D1, ILF3, NCL, S100A13, KIF20B, RAN*, and *GET4*. As we saw in the shortened 3′UTRs, the most affected genes for lengthened 3′ UTRs lie within the RNA binding function domain (GO: 0003723). MRNA processing, RNA splicing, and nucleic acid metabolic processes received the top scores, indicating a strong impact of WT tau in the regulation of mRNA isoforms at different levels. All significant enrichment terms are clustered and represented in a scatterplot in Figure 5C and Enrich-GO Cellular Component of WT Tau shortened APAs is represented in Figure 5D. In the mRNA processing domain (GO: 0006397) we identified several heterogeneous nuclear ribonucleoproteins (hnRNPs) genes (*HNRNPA3, HNRNPK, HNRNPF, HNRNPC, HNRNPDL*). HnRNPs are involved in alternative splicing, transcriptional and translational regulation, stress granules formation, cell cycle regulation, and axonal transport (Geuens et al., 2016). Their dysfunction has been shown have neurological implications, but their roles have not been comprehensively investigated. Several neurodegenerative diseases, including AD, FTD, and amyotrophic lateral sclerosis (ALS) have been associated with hnRNPs when it comes to the progression of these pathologies (Lee et al., 2013). More specifically, hnRNPK has been linked to the transcripts of several cytoskeletal genes, including *MAPT*, which is needed for axonogenesis (Liu and Szaro, 2011).

In Alzheimer’s disease, hnRNPC promotes APP translation (Lee et al., 2010) and stabilizes the APP precursors mRNA, which could suggest that increasing hnRNPC levels may promote Aβ secretion (Rajagopalan et al., 1998). Within the hnRNPs group, hnRNPA3, hnRNPF and hnRNPDL are all detected in pathological inclusions of ALS and FTD brains (Lee et al., 2013; Mori et al., 2013; Gami-Patel et al., 2016). Moreover, hnRNPK is a regulator of p53 (Low et al., 2021), which we and others recently discovered was present in elevated amounts in AD cortices (Baquero et al., 2019; Farmer et al., 2020). It has been also determined that hnRNPK sumoylation mediates p53 activity (Pelisch et al., 2012). All this evidence places hnRNPs in a central position for further experimental analysis in human brain tissues to elucidate more valuable information about the localization and function of this large family of ribonucleoproteins.

HnRNPA3 has been identified in neuronal cytoplasmic and intranuclear inclusions in patients with GGGGCC expansion repeats (Mori et al., 2013) and hnRNP F were also found to co-localize with GGGGCC expansion foci in immunoprecipitation studies (Mandler et al., 2014). In addition, western blot analyses imply that hnRNP may be in part responsible for the toxicity incurring by C9orf72 mutations, considering important RNA processes such as splicing are compromised. hnRNP A3 and K have been found associated with TDP-43 (Moujalled et al., 2017). Implications of tau-mediated APAs in hnRNPs open new venues for investigators to study new mechanistic insights of these proteins in several proteinophaties. Within RBPs group, we also observed the *MATR3* gene. This gene encodes for Matrin3, a DNA/RNA-binding protein. Mutations in this gene cause familial ALS/FTD, and MATR3 pathology is a feature of sporadic disease, suggesting that its dysfunction is inherently linked to ALS pathogenesis (Malik et al., 2018).

Shorter 3′UTR are generally associated with enhanced translation of the mRNA APA in the presence of WT tau, which supports the finding that high-levels of hnRNPs sustain dysfunction of stress granules in ALS and FTD. Recent proteomic analysis in AD human Neurofibrillary Tangles (NFTs) showed that phospho-tau in NFTs is associated with more than 500 proteins (Drummond et al., 2020). We observed several of these proteins in the APAs shortened WT tau, such as HNRNPK, ILF3, AP2B1, RAN, RAB11A, HSP90B1, PARP1, PPIA, NCL, HNRNPA3, HSP90AA1, HNRNPC, MATR3 (Figure 5E) and EMB (Figure 5F). It is intriguing that the presence of chaperone Hsp90, a tau-regulated gene, plays a crucial role in neurodegenerative pathologies and has been studied in AD or a long time (Campanella et al., 2018).

These observations suggest that tau has early effects on gene expression that results in later stages of toxic associations commonly found in neurodegeneration. Enrich-GO (Cellular Function) of shortened-APAs genes by WT tau is provided in “Supplemental Information” section. GO-Cellular Process, Molecular Process and Cellular Components bar charts of shortened APAs are shown in Supplementary Figure 4.

### SWI/SFN, THO Complexes, and Several RNA-Binding Protein Precursors Display Lengthened 3′UTRs in Presence of WT Tau

**FIGURE 6:**
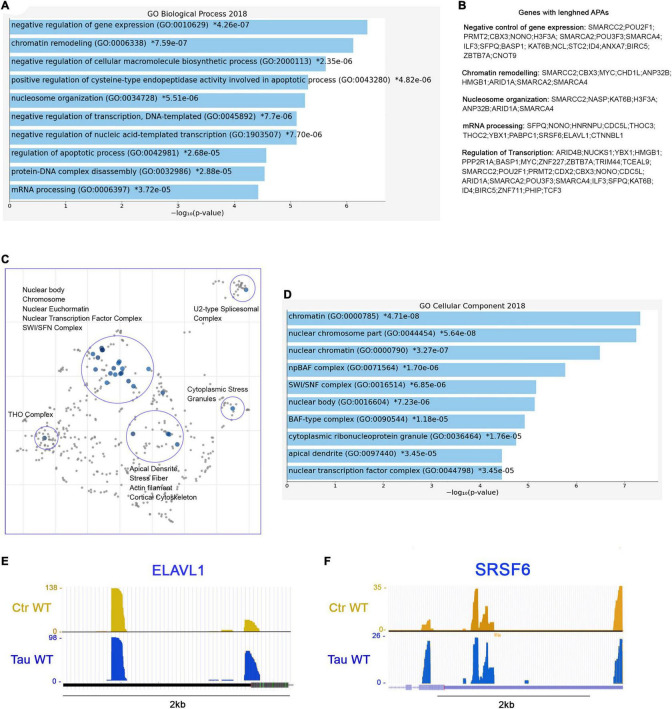
RNA processing and splicing precursor’s display lengthened APAs in presence of WT tau. **(A)** Enrich-GO Biological Process of WT tau lengthened APAs (*p*-value reported). **(B)** Partial list of biological process genes with lengthened APAs upon presence of WT tau negative control of gene expression, chromatin remodeling, nucleosome organization, mRNA processing and regulation of transcription). **(C)** The Cellular Components scatterplot of lengthened APAs in WT tau is organized so that similar gene sets are clustered together. The larger blue points represent significantly enriched terms—the darker the blue, the more significant the term and the smaller the *p*-value. The gray points are not significant. Plots has been generated and downloaded using scatter plot visualization Appyter. **(D)** Enrich-GO Cellular Component of WT tau lengthened APAs (*p*-value reported). Representative poly-A site usage for lengthened poly-A tails from PAC-Seq of ELAVL **(E)** and SRSF6 **(F)** transcripts. An asterisk (*) next to a *p*-value indicates the term also has a significant adjusted *p*-value (<0.05).

Further analysis revealed 173 genes with lengthened APAs. The complete list of the 173 genes with lengthened APAs is reported in “Supplemental Information” section. Among these genes, we found that many of them are related to three major biological process: chromatin remodeling (GO: 0006338), negative regulation of gene expression (GO: 0010629), and mRNA processing (GO: 0006397) (Figure 6A). To be more specific, we noticed several genes belonging to the ATP-dependent chromatin remodeling complex npBAF (mammalian SWI/SFN, GO: 0071564): *SMARCC2*, *ARID1A*, *SMARCA2*, and *SMARCA4*. This complex is found in neuronal progenitor’s cells and post-mitotic neurons, and it is essential for the maturation of the post-mitotic neuronal phenotype as well as long-term memory formation (Sokpor et al., 2017). Along with the chromatin remodeling complex, other genes contained altered APAs, including pericentric chromatin components (GO: 0005721, *HELLS*, and *CBX3*), and nuclear chromatin factors (GO: 0000790, *SMARCC2, CBX3, H3F3A, NUCKS1, ARID1A, SMARCA2, SMARCA4, HIST2H2AC, RAD50, NASP, MYC, NSMF, TCF3*) (Figure 6B).

Several nuclear speck (GO: 0016607) genes were also identified: *BASP1, POM,; ERBI, YLPM1, HNRNPU, LUC7L3, CDC5L, TCF3, SRSF6* (Figure 6F), and *KIF20B.* Cytoplasmic ribonucleoprotein granule (GO: 0036464) and cytoplasmic stress granules (GO: 0010494) genes were delineated as *MBNL1, CARHSP1, NCL, HNRNPU, IQGAP1, YBX1, RAC1, PABPC1, CNOT9*. Within the domain of RNA processing, two genes *THOC2* and *THOC3* were also identified. They are components of the THO complex (GO: 0000445) involved in efficient export of poly-adenylated RNA and spliced RNAs (Stewart, 2019).

The THO complex appears to coordinate transcripts for synapses development and dopamine neuron survival (Maeder et al., 2018). Recently, it has been found to interact with ZC3H14, which regulates the processing of neuronal transcripts (Morris and Corbett, 2018), so it is not surprising to find in our dataset another polyadenosine RNA-binding protein *ZC3H15* on the list of lengthened APAs. These observations indicate that export complex RNA precursors are meaningfully affected by WT tau.

Not surprisingly, many translation initiation factors (GO: 0003743) were also discovered in our analysis including *EIF2S3, EIF3E, EIF3A, EIF1*, and *EIF4G1.* It is important to note that many APA-lengthened proteins in our study are RNA-Binding Proteins (RBPs). In fact, 46/173, or ∼27% of the total were. RBPs are implicated in the pathogenesis and progression of numerous neurodegenerative diseases, and they are linked to toxic interactions and aggregations in amyloidogenic proteins such Amyloid-beta and tau. The subsequent dysfunction of RBPs is closely related to distinct pathways that are altered in proteinophaties (Maziuk et al., 2017).

Considering the above, we also studied the presence of lengthened APAs of *ELAVL1* (Figure 6E). This gene encodes for HuR (RBPs), which is a neuroprotective protein. This protein has been demonstrated in the regulation of oxidative metabolism in neurons as a way to protect from neurodegeneration (Skliris et al., 2015).

Apical dendrites (GO: 0097440) (*MAP1B, NSMF and CLU*) and other cytoskeletal genes (*ACTR2, LIMA1, TPM4, PPP2R1A, BASP1, TARS, PHIP, NSMF, IQGAP1, RAC1, CLU*, and *SMARCA2*) display lengthened poly-A tails as well. Enrich-GO (Cellular Function) of lengthened-APAs genes in WT tau is provided in “Supplemental Information” section. GO-Cellular Process, Molecular Process and Cellular Components bar charts of lengthened-APAs are shown in Supplementary Figure 4.

### P301L Tau Modulates 3′UTRs of RNA Export Complex *THOC* and Splicing Precursors *SNRPE*

**FIGURE 7:**
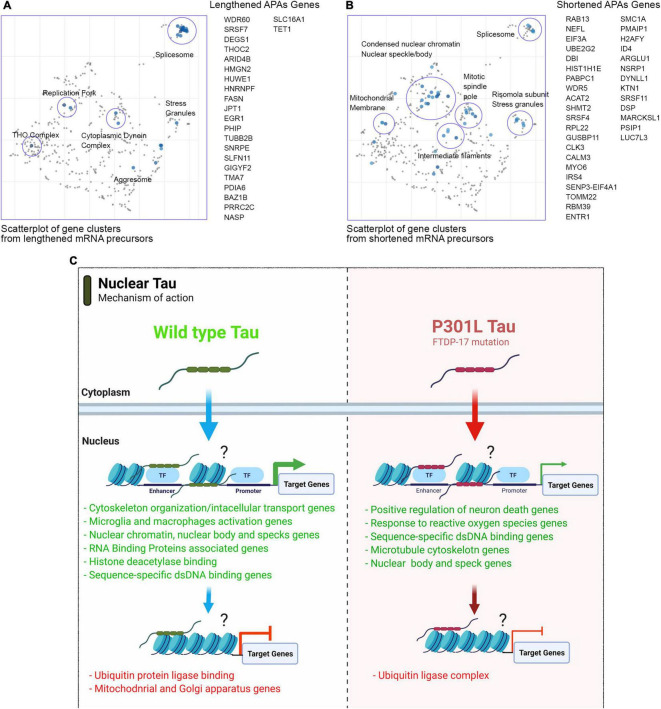
Mutant P301L tau modulates APAs associated with spliceosome and nuclear chromatin. **(A)** Scatterplot of gene clusters from lengthened mRNA precursors upon P301L Tau expression. **(B)** Scatterplot of gene clusters from shortened mRNA precursors upon P301L Tau expression. **(C)** Model for nuclear tau activity to transcriptional and post-transcriptional levels.

In P301L tau precursor APAs, we detected 23 lengthened genes in total. More specifically, the THOC2 gene, which is a component of the THO complex (GO: 0000445) was lengthened in WT tau. Another gene of the small nuclear ribonucleoprotein complex (SNRPE) was detected. *SNRPE* is also a gene for the spliceosome complex (GO: 0005681) (Figure 7A). Lastly, the nuclear replication fork (GO: 0043596) gene *BAZ1B* was also observed.

In contrast to WT tau, P301L tau induces lengthening of the *HNRNPF* gene. HnRNPs represent a large RNA-Binding protein family that contributes to many aspects of nucleic acid metabolism, including alternative splicing, mRNA stabilization, transcriptional, and translational regulation (Geuens et al., 2016). Dysregulation of RNA metabolism is crucial in the pathogenesis of several neurodegenerative diseases as Parkinson’s (Lu et al., 2014), FTD and overlaps with aspects of ALS. Some studies revealed possible involvement of hnRNPs in the pathogenesis and progression of these diseases (Bampton et al., 2020). Furthermore, hnRNP F has been uncovered in RNA foci in human brain tissue of FTD-ALS patients (Lee et al., 2013). Affinity pull-down assays and genome-wide analysis also revealed a hnRNP F-bound splicing complex that regulates neuronal and oligodendroglial differentiation pathways in the developing brain (Mandler et al., 2014). As observed for WT tau, the mutant P301L form also modulates several RNA-Binding Proteins (GO: 0003723): *SLFN11, HNRNPF, FASN, HUWE1, PRRC2C, THOC2, HMGN2, SRSF7*, and *GIGYF7.* We found 34 genes in total with evidence of APA and shortened 3′UTRs (Figure 7B). The three top-scored cellular components were nuclear speck (GO: 0016607), nuclear body (GO: 0016604) with *RBM39* (ALS associated gene; Couthouis et al., 2011) and Nuclear heterochromatin genes (GO: 0005720). Nuclear speak and body genes consisted of *LUC7L3, SRSF4, NSRP1, and SRSF11*. Nuclear heterochromatin genes detected were *H2AFY* and *HIST1H1E*. *H2AFY* encodes for a variant of the H2A histone that is present in a subset of nucleosomes where its role is to represses transcription (Doyen et al., 2006).

The Cellular Components scatterplot of lengthened APAs in WT Tau is presented in Figure 6C and GO Cellular component bar charts in Figure 6D.

These data suggest that the mutant P301L form of tau reduces activity in transcription and alternative poly(A) tails processes due to loss-of-function. However, P301L tau does generate different mRNA isoforms of transcripts mainly translated in splicing factors, nuclear speckle/body structures and chromatin remodeling proteins. Enrich-GO (Cellular Function) of shortened and lengthened-APAs by P301L tau is provided in “Supplemental Information” section. GO-Cellular Process, Molecular Process and Cellular Components bar charts of shortened and lengthened-APAs are shown in Supplementary Figure 5.

## Discussion

In this study, we revealed new mechanistic insights into non-canonical tau functions. In particular, we showed novel tau activities in transcription and alternative poly-adenylation (APA) pathways. APA is a widespread mechanism of gene regulation that generates 3′ ends in transcripts made by RNA polymerase II (Tian and Manley, 2013). APA is regulated in cell proliferation, differentiation and extracellular cues. It occurs in the 3′UTR and leads to the production of mRNA isoforms, followed by splicing which leads to the production of distinct protein isoforms (Tian and Manley, 2013). Tau is typically described as an abundant neuronal microtubule-binding protein. Recently, we observed its presence within non-neuronal human cell lines and neuronal nuclei in AD brains (Montalbano et al., 2019, 2020) alongside other study (Violet et al., 2014). We were particularly interested in the possibility of non-canonical tau functions. We hypothesized that nuclear tau acts as a transcriptional regulator. To test our hypothesis, we used the tau inducible HEK system, which is a well-established cell line capable of studying mechanisms related to the tau aggregation process within a controlled system of MAPT gene expression (Koren et al., 2019). Our study employed new technologies such as Poly(A)-ClickSeq to resolve whether genes were upregulated or downregulated by WT and P301L tau in an *in vitro* model. Furthermore, we analyzed alternative polyadenylation (APA) profiles under the presence of WT and P301L tau (Couthouis et al., 2011).

Our results suggest that both WT and P301L tau are able to shuttle into the nuclei (Figure 4). This observation confirmed our previous observations (Montalbano et al., 2019). We did not investigate the effect of the P301L mutation on nuclei-cytoplasm shuttling in this report. The decreased number of genes expressed in P301L cells suggests that this particular mutation of tau impairs transcriptional activity. We did not investigate the consequences of P301L tau in great detail, but our observations suggest new mechanistic insights linked to alternative nuclear tau function.

One APA transcript of significance is the *SFPQ* gene, which we identified in WT tau expression as having a lengthened 3′UTR. *SFPQ* has been associated with tau as a critical factor for rapid progression of AD, and it has been observed as downregulated in post-mortem brain tissue of rapidly progressive AD patients (Younas et al., 2020). Therefore, the lengthened APAs in this gene could explain the down-regulation in the presence of a high level of tau, which mimics late-stage AD. *In vitro* data of SFPQ down-regulation due to human tau suggest a causal role of tau, possibly through the alternative poly-adenylation of *SFPQ* transcripts.

Further analysis comparing 3′UTRs lengthened between WT and P301L tau revealed that a significant number of RBPs showed lengthened 3′UTRs in P301L compared to WT tau. For example, we detected 72 RBPs including *FUS* (found in Supplemental Information section). These data suggest a significant difference in RNA isoforms based on genetic tau background, which then subsequently modulates different aspects of RNA metabolism in neurons.

Using the same cellular models, we determined that the prominent form of nuclear tau is monomeric, but Tet induction causes tau oligomerization within the nuclei (Montalbano et al., 2019). The formation of large and nuclear oligomeric forms is another possible explanation observed as a consequence of mutated tau. Mutant P301L tau shows a distinct aggregation mechanism compared to WT (Strang et al., 2018) and aggregates faster than WT (Barghorn et al., 2000; Aoyagi et al., 2007). For example, monomeric tau in the cytoplasm of cells producing (WT or P301L) tau aggregate and subsequently avoid nuclear translocation. In addition, aggregation in the cytoplasm and within the nuclei of tau reduces the pool of monomeric nuclear tau. This pathological mechanism can compete with functional monomeric and oligomeric tau, which then alters tau transcriptional activity. This phenomenon should be investigated in the near future using neuronal models. Another function of tau is binding DNA *in vitro*. Overall, the multifunctional nature of nuclear tau should be thoroughly scrutinized in order to identify unrevealed functions connected to DNA expression and RNA processing. We suggest that the nature of nuclear tau as a transcriptional factor, chromatin remodeler and/or transcriptional co-factor must be elucidated using proper models such as induced pluripotent stem cells or mouse primary neurons carrying mutation on P301 site. At this stage, we can only hypothesize the direct and indirect effects of tau during transcription.

This study utilized PAC-ClickSeq technology to identify the APA modulated by P301L and WT tau. Alternative Poly-A (APA) sites in human genome have been identify mainly in 3′UTRs (UTR-APA) sites, which harbor diverse regulatory sequences. This type of APA can change the length and composition of 3′UTR, which subsequently affects the binding of miRNAs and/or RBPs. This post-transcriptional modification leads to differences in mRNA stability, export, localization, translational efficiency (Gruber and Zavolan, 2019). Although the currently accepted theory is that genes with longer 3′UTR tend to show decreased expression levels, this does not necessarily mean that every single gene with a longer 3′UTR is less stable those with a shorter one.

We plan to investigate these findings using primary neurons and *in vivo* models in the near future. We are choosing these alternative models because the iHEK cell model have inherent limitations in terms of reliability as a neuronal system. However, the iHEK cells used in this study are an established model used by many researchers to study the mechanistic insights of tau aggregation and toxicity. The results presented in this study support non-canonical functions of tau. Therefore, we report broad tau-driven, post-transcriptional regulation in APAs by both WT and P301L tau considering both cell lines produced high levels of monomeric and aggregated tau. In this study, we did not investigate which tau isoform regulates APA in cells and by what method tau regulates APAs, but we established a new category of interest in post-translational modification. We hope further studies of nuclear tau and its relation to DNA and RNA processing will identify new targets in tauopathies and eventually find new therapeutic targets.

### Limitations of the Study

As mentioned in the discussion, the main limitation of this study is the nature of tau inducible HEK cells. We are aware that further study on neuronal cells is necessary. However, iHEK models are commonly used to study mechanisms that are tau-dependent and several of them have been translated into neurons models. All relevant datasets used and/or analyzed in this current study are available upon request from the corresponding author.

### Supplemental Information

The source data underlying all main and Supplementary Figures are provided as a Source Data file. RNAseq datasets is uploaded to NCBI SRA, reference number: PRJNA744518. Figures 1A, 4F, 7C were generated using BioRender Software^1^.

## Author Summary

While tau biology has been extensively studied and closely linked to several neurodegenerative diseases, our current understanding of tau’s functions in the nucleus is limited. Given the role of tau in disease progression and pathogenesis, elucidating the function of tau activity in transcription and its nuclear accumulation may reveal novel therapeutic targets; therefore, helping identify new upstream pathways that have yet to be investigated. In this study, we used tau-inducible cell lines to uncover new molecular mechanisms by which tau functions in the nucleus. This study systematically investigates the changes in transcriptomic and alternative polyadenylation profiles modulated by WT and mutant P301L tau protein. In this manuscript, we report following new findings (**i**) tau modulates gene expression of transcripts associated with chromatin remodeling and splicing complexes; (**ii**) WT and mutant P301L tau regulate, differentially, transcription and alternative polyadenylation (APA) profiles; and (**iii**) P301L mutation affects the transcription mediated by tau protein. The potential role of tau in mediating transcription and alternative polyadenylation processes is not well studied, representing a novelty in the field. Therefore, this research establishes a new direction for investigating tau nuclear function in both human and mouse brains.

## Data Availability Statement

The datasets presented in this study can be found in online repositories. The names of the repository/repositories and accession number(s) can be found in the article/Supplementary Material.

## Author Contributions

MM, AR, and RK: conceptualization and methodology. MM, EJ, SM, AE, and SG: investigation. AR and EJ: transcriptomic analysis. MM: writing—original draft. RK: funding acquisition and resources. MM and RK: supervision. All authors: writing—review and editing.

## Conflict of Interest

The authors declare that the research was conducted in the absence of any commercial or financial relationships that could be construed as a potential conflict of interest.

## Publisher’s Note

All claims expressed in this article are solely those of the authors and do not necessarily represent those of their affiliated organizations, or those of the publisher, the editors and the reviewers. Any product that may be evaluated in this article, or claim that may be made by its manufacturer, is not guaranteed or endorsed by the publisher.

## Footnotes

1
https://biorender.com

## Abbreviations

AD: Alzheimer’s disease
APA: alternative polyadenylation
DDR: DNA damage response
FTD: frontal temporal dementia
GO: Gene Ontology
GSEA: gene set enrichment analysis
MTs: microtubules
PAC: poly(A) cluster
PAC-Seq: Poly(A)-ClickSeq, PAP, poly-A polymerase
PAS: poly(A) site
RNA: ribonucleotide acid
Tet: Tetracycline; 3′
UTR: 3′ untranslated region
iHEK: inducible human embryonic kidney cells
ER: endoplasmic reticulum.
