# Supplementary material for: Tau Modulates mRNA Transcription, Alternative Polyadenylation Profiles of hnRNPs, Chromatin Remodeling and Spliceosome Complexes
Source: Front Mol Neurosci. 2021 Dec 3;14:742790. doi: 10.3389/fnmol.2021.742790 (PMC8678415; doi:10.3389/fnmol.2021.742790)
Supplement: Supplementary file 1 [file Data_Sheet_1.PDF]

Supplemental Information

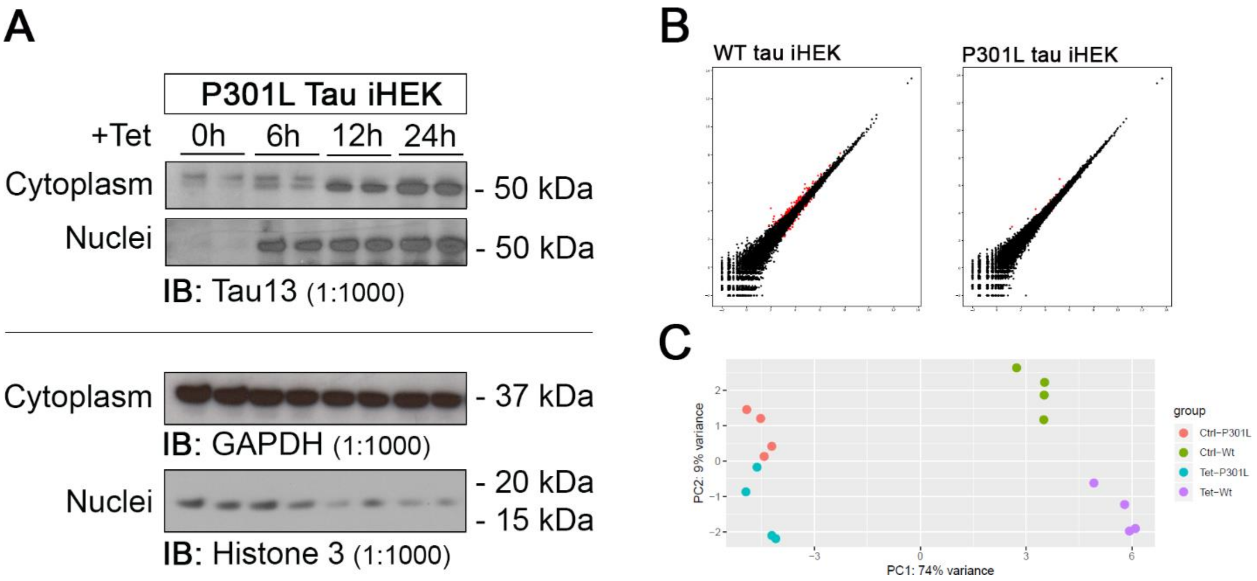

Fig S1. Cytoplasmatic and nuclear P301L tau protein levels. (**A**) WB of cytoplasmic and nuclear fractions of P301L Tau iHEK at 0, 6, 12 and 24h after Tet induction. Samples are presented in technical duplicates. Immunoblot (IB) with following antibodies are presented: Tau 13 (1:1000). Cytoplasm and Nuclear fractions loading control GAPDH (1:1000) and Histone 3 (1:1000) has been used, respectively. (**B**) RNA-Seq scatterplots from WT and P301L Tau iHEK are represented; red spots represent statistically significant genes with large fold changes. (**C**) Principal component (PC) analysis showed a higher difference in RNA expression profiles in presence of WT Tau compared to the P301L Tau group.

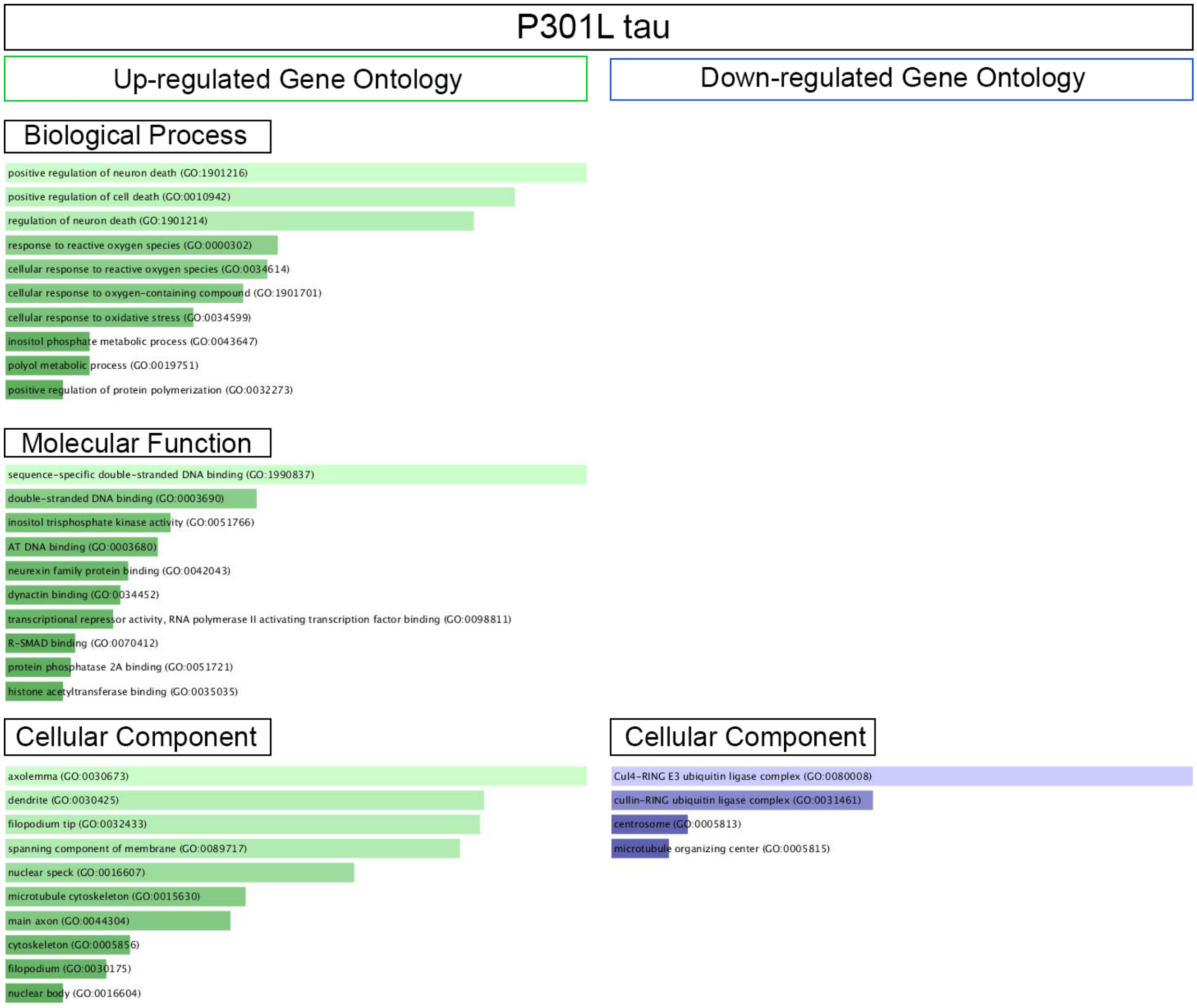

Fig S2. Up- and Down regulated genes in P301L Tau Gene Ontology. Left Column (Green) Up-regulated genes analyzed by Enrich-GO and divided by Biological Process, Molecular Function and Cellular Component. Right Column (Blue) Down-regulated genes analyzed by Enrich-GO and divided by Biological Process, Molecular Function and Cellular Component. Biological component and Molecular component of down-regulated gene (DCAF12) did not detect significant value and no bar graph are reported.

A. GO-Chromatin organization

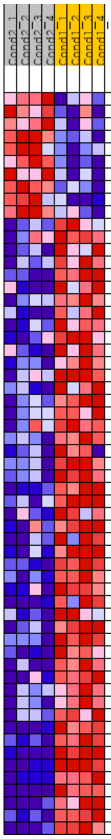

SampleName		
MYC	MYC	"MYC proto-oncogene, bHLH transcription factor [Source:HGNC Symbol;Acc:HGNC:7553]"
MCM2	MCM2	minichromosome maintenance complex component 2 [Source:HGNC Symbol;Acc:HGNC:6944]
CENPV	CENPV	centromere protein V [Source:HGNC Symbol;Acc:HGNC:29920]
DNAJC2	DNAJC2	DnaJ heat shock protein family (Hsp40) member C2 [Source:HGNC Symbol;Acc:HGNC:13192]
HMGN5	HMGN5	high mobility group nucleosome binding domain 5 [Source:HGNC Symbol;Acc:HGNC:8013]
HMGB2	HMGB2	high mobility group box 2 [Source:HGNC Symbol;Acc:HGNC:5000]
NUDT5	NUDT5	nudix hydrolase 5 [Source:HGNC Symbol;Acc:HGNC:8052]
SKP1	SKP1	S-phase kinase associated protein 1 [Source:HGNC Symbol;Acc:HGNC:10899]
MKI67	MKI67	marker of proliferation Ki-67 [Source:HGNC Symbol;Acc:HGNC:7107]
HMGA1	HMGA1	high mobility group AT-hook 1 [Source:HGNC Symbol;Acc:HGNC:5010]
HMGB1	HMGB1	high mobility group box 1 [Source:HGNC Symbol;Acc:HGNC:4983]
HNRNPU	HNRNPU	heterogeneous nuclear ribonucleoprotein U [Source:HGNC Symbol;Acc:HGNC:5048]
ACTB	ACTB	actin beta [Source:HGNC Symbol;Acc:HGNC:132]
FBL	FBL	fibrillarin [Source:HGNC Symbol;Acc:HGNC:3599]
HDAC2	HDAC2	histone deacetylase 2 [Source:HGNC Symbol;Acc:HGNC:4853]
GPX4	GPX4	glutathione peroxidase 4 [Source:HGNC Symbol;Acc:HGNC:4556]
NASP	NASP	nuclear autoantigenic sperm protein [Source:HGNC Symbol;Acc:HGNC:7644]
SRPK1	SRPK1	SRSF protein kinase 1 [Source:HGNC Symbol;Acc:HGNC:11305]
CHD4	CHD4	chromodomain helicase DNA binding protein 4 [Source:HGNC Symbol;Acc:HGNC:1919]
HP1BP3	HP1BP3	heterochromatin protein 1 binding protein 3 [Source:HGNC Symbol;Acc:HGNC:24973]
NAP1L4	NAP1L4	nucleosome assembly protein 1 like 4 [Source:HGNC Symbol;Acc:HGNC:7640]
MCM3AP	MCM3AP	minichromosome maintenance complex component 3 associated protein [Source:HGNC Symbol;Acc:HGNC:6946]
TPR	TPR	"translocated promoter region, nuclear basket protein [Source:HGNC Symbol;Acc:HGNC:12017]"
TRIP12	TRIP12	thyroid hormone receptor interactor 12 [Source:HGNC Symbol;Acc:HGNC:12306]
SMARCE1	SMARCE1	"SWI/SNF related, matrix associated, actin dependent regulator of chromatin, subfamily e, member 1 [Source:HGNC Symbol;Acc:HGNC:11109]"
SMARCA5	SMARCA5	"SWI/SNF related, matrix associated, actin dependent regulator of chromatin, subfamily a, member 5 [Source:HGNC Symbol;Acc:HGNC:11101]"
ATAD2	ATAD2	ATPase family AAA domain containing 2 [Source:HGNC Symbol;Acc:HGNC:30123]
HMGN1	HMGN1	high mobility group nucleosome binding domain 1 [Source:HGNC Symbol;Acc:HGNC:4984]
SETD2	SETD2	"SET domain containing 2, histone lysine methyltransferase [Source:HGNC Symbol;Acc:HGNC:18420]"
CHAF1A	CHAF1A	chromatin assembly factor 1 subunit A [Source:HGNC Symbol;Acc:HGNC:1910]
SMARCC1	SMARCC1	"SWI/SNF related, matrix associated, actin dependent regulator of chromatin subfamily c member 1 [Source:HGNC Symbol;Acc:HGNC:11104]"
HNRNPC	HNRNPC	heterogeneous nuclear ribonucleoprotein C [Source:HGNC Symbol;Acc:HGNC:5035]
AKAP8	AKAP8	A-kinase anchoring protein 8 [Source:HGNC Symbol;Acc:HGNC:378]
EP400	EP400	E1A binding protein p400 [Source:HGNC Symbol;Acc:HGNC:11958]
AKAP8L	AKAP8L	A-kinase anchoring protein 8 like [Source:HGNC Symbol;Acc:HGNC:29857]
MBD2	MBD2	methyl-CpG binding domain protein 2 [Source:HGNC Symbol;Acc:HGNC:6917]
SAFB	SAFB	scaffold attachment factor B [Source:HGNC Symbol;Acc:HGNC:10520]
SFPQ	SFPQ	splicing factor proline and glutamine rich [Source:HGNC Symbol;Acc:HGNC:10774]
JMJD1C	JMJD1C	jumonji domain containing 1C [Source:HGNC Symbol;Acc:HGNC:12313]
SETD5	SETD5	SET domain containing 5 [Source:HGNC Symbol;Acc:HGNC:25566]
MAP1S	MAP1S	microtubule associated protein 1S [Source:HGNC Symbol;Acc:HGNC:15715]
PRMT2	PRMT2	protein arginine methyltransferase 2 [Source:HGNC Symbol;Acc:HGNC:5186]
KDM5B	KDM5B	lysine demethylase 5B [Source:HGNC Symbol;Acc:HGNC:18039]
PAXBP1	PAXBP1	PAX3 and PAX7 binding protein 1 [Source:HGNC Symbol;Acc:HGNC:13579]
BRD9	BRD9	bromodomain containing 9 [Source:HGNC Symbol;Acc:HGNC:25818]
BRD3	BRD3	bromodomain containing 3 [Source:HGNC Symbol;Acc:HGNC:1104]
HIRA	HIRA	histone cell cycle regulator [Source:HGNC Symbol;Acc:HGNC:4916]
SFMBT1	SFMBT1	Scm like with four mbt domains 1 [Source:HGNC Symbol;Acc:HGNC:20255]
RNF168	RNF168	ring finger protein 168 [Source:HGNC Symbol;Acc:HGNC:26661]
MSL3	MSL3	MSL complex subunit 3 [Source:HGNC Symbol;Acc:HGNC:7370]
SUDS3	SUDS3	"SDS3 homolog, SIN3A corepressor complex component [Source:HGNC Symbol;Acc:HGNC:29545]"
BAZ2A	BAZ2A	bromodomain adjacent to zinc finger domain 2A [Source:HGNC Symbol;Acc:HGNC:962]
POLE3	POLE3	"DNA polymerase epsilon 3, accessory subunit [Source:HGNC Symbol;Acc:HGNC:13546]"
ANP32E	ANP32E	acidic nuclear phosphoprotein 32 family member E [Source:HGNC Symbol;Acc:HGNC:16673]
PRDM2	PRDM2	PR/SET domain 2 [Source:HGNC Symbol;Acc:HGNC:9347]
ANP32B	ANP32B	acidic nuclear phosphoprotein 32 family member B [Source:HGNC Symbol;Acc:HGNC:16677]
CENPT	CENPT	centromere protein T [Source:HGNC Symbol;Acc:HGNC:25787]
KAT7	KAT7	lysine acetyltransferase 7 [Source:HGNC Symbol;Acc:HGNC:17016]
PTMA	PTMA	prothymosin alpha [Source:HGNC Symbol;Acc:HGNC:9623]

B. GO-Chromatin remodeling

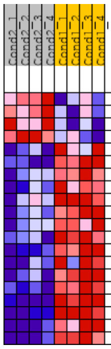

SampleName		
MYC	MYC	"MYC proto-oncogene, bHLH transcription factor [Source:HGNC Symbol;Acc:HGNC:7553]"
CENPV	CENPV	centromere protein V [Source:HGNC Symbol;Acc:HGNC:29920]
NUDT5	NUDT5	nudix hydrolase 5 [Source:HGNC Symbol;Acc:HGNC:8052]
HMGA1	HMGA1	high mobility group AT-hook 1 [Source:HGNC Symbol;Acc:HGNC:5010]
ACTB	ACTB	actin beta [Source:HGNC Symbol;Acc:HGNC:132]
HDAC2	HDAC2	histone deacetylase 2 [Source:HGNC Symbol;Acc:HGNC:4853]
NASP	NASP	nuclear autoantigenic sperm protein [Source:HGNC Symbol;Acc:HGNC:7644]
CHD4	CHD4	chromodomain helicase DNA binding protein 4 [Source:HGNC Symbol;Acc:HGNC:1919]
TPR	TPR	"translocated promoter region, nuclear basket protein [Source:HGNC Symbol;Acc:HGNC:12017]"
SMARCE1	SMARCE1	"SWI/SNF related, matrix associated, actin dependent regulator of chromatin, subfamily e, member 1 [Source:HGNC Symbol;Acc:HGNC:11109]"
SMARCA5	SMARCA5	"SWI/SNF related, matrix associated, actin dependent regulator of chromatin, subfamily a, member 5 [Source:HGNC Symbol;Acc:HGNC:11101]"
SMARCC1	SMARCC1	"SWI/SNF related, matrix associated, actin dependent regulator of chromatin subfamily c member 1 [Source:HGNC Symbol;Acc:HGNC:11104]"
HNRNPC	HNRNPC	heterogeneous nuclear ribonucleoprotein C [Source:HGNC Symbol;Acc:HGNC:5035]
MBD2	MBD2	methyl-CpG binding domain protein 2 [Source:HGNC Symbol;Acc:HGNC:6917]
KDM5B	KDM5B	lysine demethylase 5B [Source:HGNC Symbol;Acc:HGNC:18039]
BAZ2A	BAZ2A	bromodomain adjacent to zinc finger domain 2A [Source:HGNC Symbol;Acc:HGNC:962]
POLE3	POLE3	"DNA polymerase epsilon 3, accessory subunit [Source:HGNC Symbol;Acc:HGNC:13546]"
ANP32E	ANP32E	acidic nuclear phosphoprotein 32 family member E [Source:HGNC Symbol;Acc:HGNC:16673]
CENPT	CENPT	centromere protein T [Source:HGNC Symbol;Acc:HGNC:25787]
PTMA	PTMA	prothymosin alpha [Source:HGNC Symbol;Acc:HGNC:9623]

C. GO-Covalent Chromatin Modification

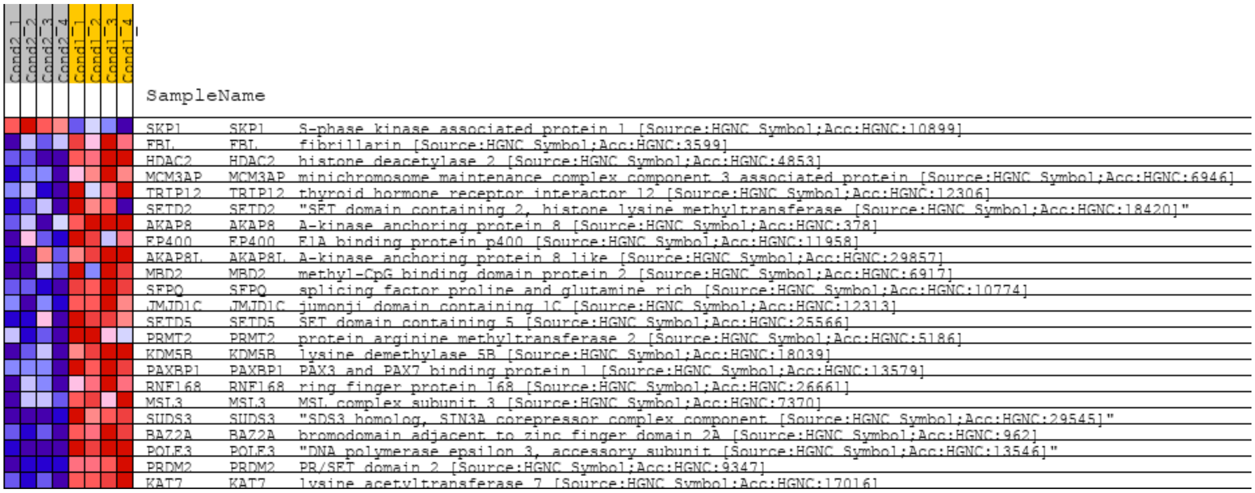

D. GO-Histone Binding

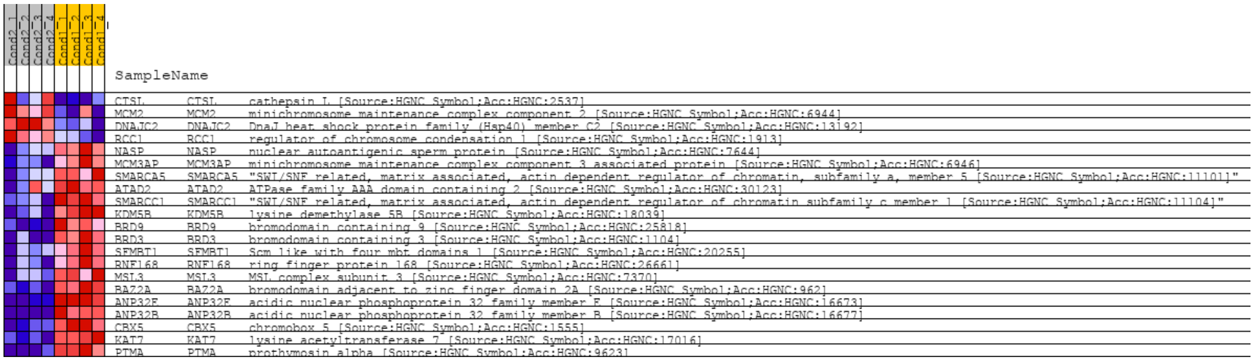

E. GO-Nucleosome Organization

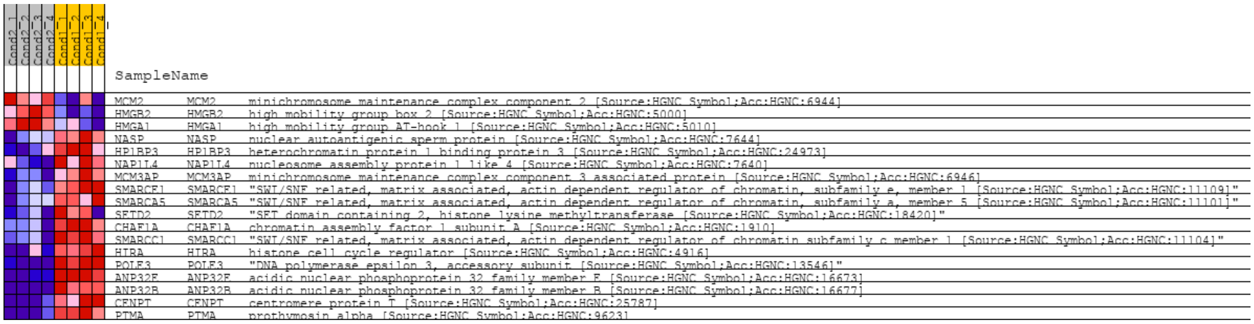

F. P301L Tau iHEK cell fraction WB

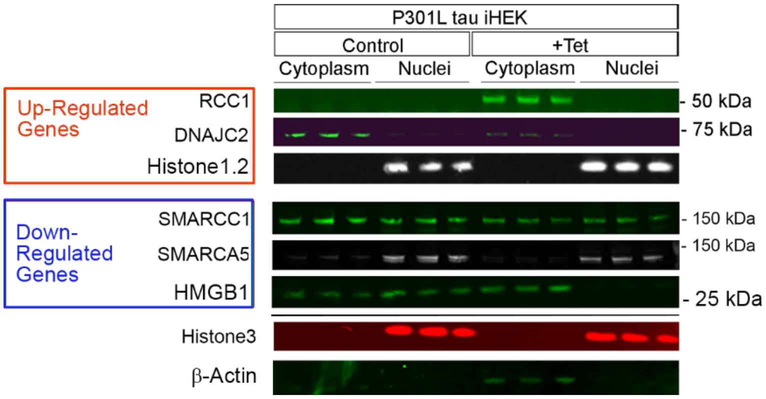

Fig S3. Heat maps of up- and down-regulated genes by WT tau. (**A**) GSEA GO-Chromatin organization heat map in WT Tau. (**B**) GSEA GO-Chromatin remodeling heat map in WT Tau. (**C**) GSEA GO-Covalent Chromatin Modification heat map in WT Tau. (**D**) GSEA GO-Histone Binding heat map in WT Tau. (**E**) GSEA GO-Nucleosome Organization heat map in WT Tau. Four replicates are represented for each condition (-Tau (UT) and +Tau), blue grades represent down-regulated genes and red grades represent up-regulated genes. (**F**) IB of RCC1, DNAJC2, Histone 1.2, SMARCC1, SMARCA5, HMGB1 in P301L tau iHEK cell fractions. Histone3 and β-Actin have been used for nuclear and cytoplasmatic fractions purity controls, respectively.

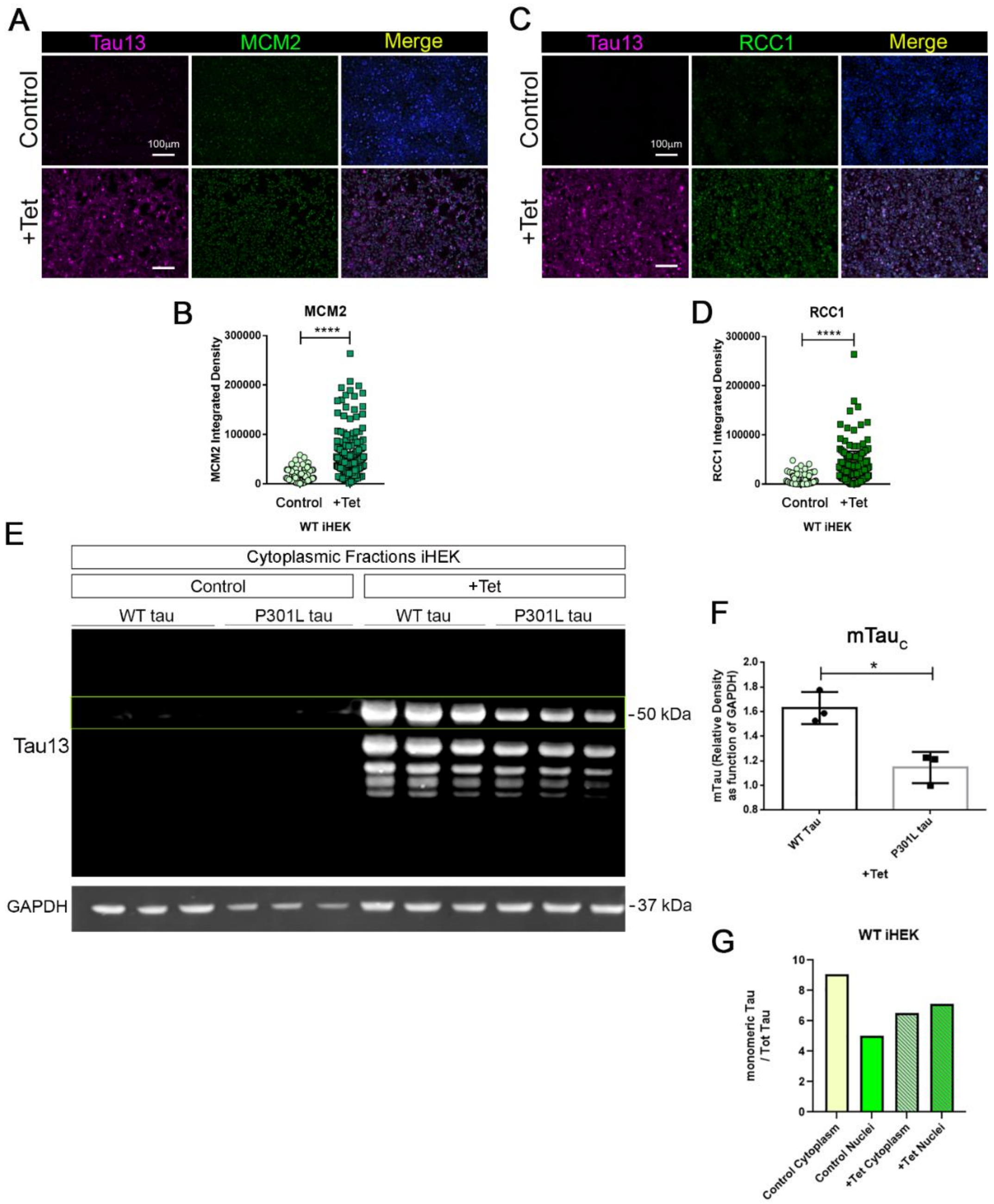

Fig S4. Validation of MCM2, RCC1 and cytoplasmic tau expression in iHEK cell lines. (**A**) Representative Immuno-fluorescence of control and +Tet Tau iHEK stained with Tau13 (magenta) and MCM2 (green), magnification 20x white scale bar: 100µm. (**B**) Plot of MCM2 integrated density in WT iHEK (unpaired t-test, Control vs +Tet, *p<0.0001*, ****). (**C**) Representative Immuno-fluorescence of control and +Tet Tau iHEK stained with Tau13 (magenta) and RCC1 (green), magnification 20x white scale bar: 100µm. (**D**) Plot of RCC1 integrated density in WT iHEK (unpaired t-test, Control vs +Tet, *p<0.0001*, ****). (**E**) Tau13 IB of Untreated (left 1-6 lines) and Tet induced (7-12 lines) of cytoplasmic fractions from WT and P301L Tau iHEK. GAPDH IB was used as loading control. (**F**) Bar graphs represent monomeric Tau (green box) in the cytoplasm quantified and normalized as function of GAPDH. Unpaired t-test, WT tau vs P301L tau *p<0.05*, *. (**G**) Ratio between Monomeric Tau and Total Tau in cytoplasmatic and nuclear fractions of Control and +Tet WT tau iHEK.

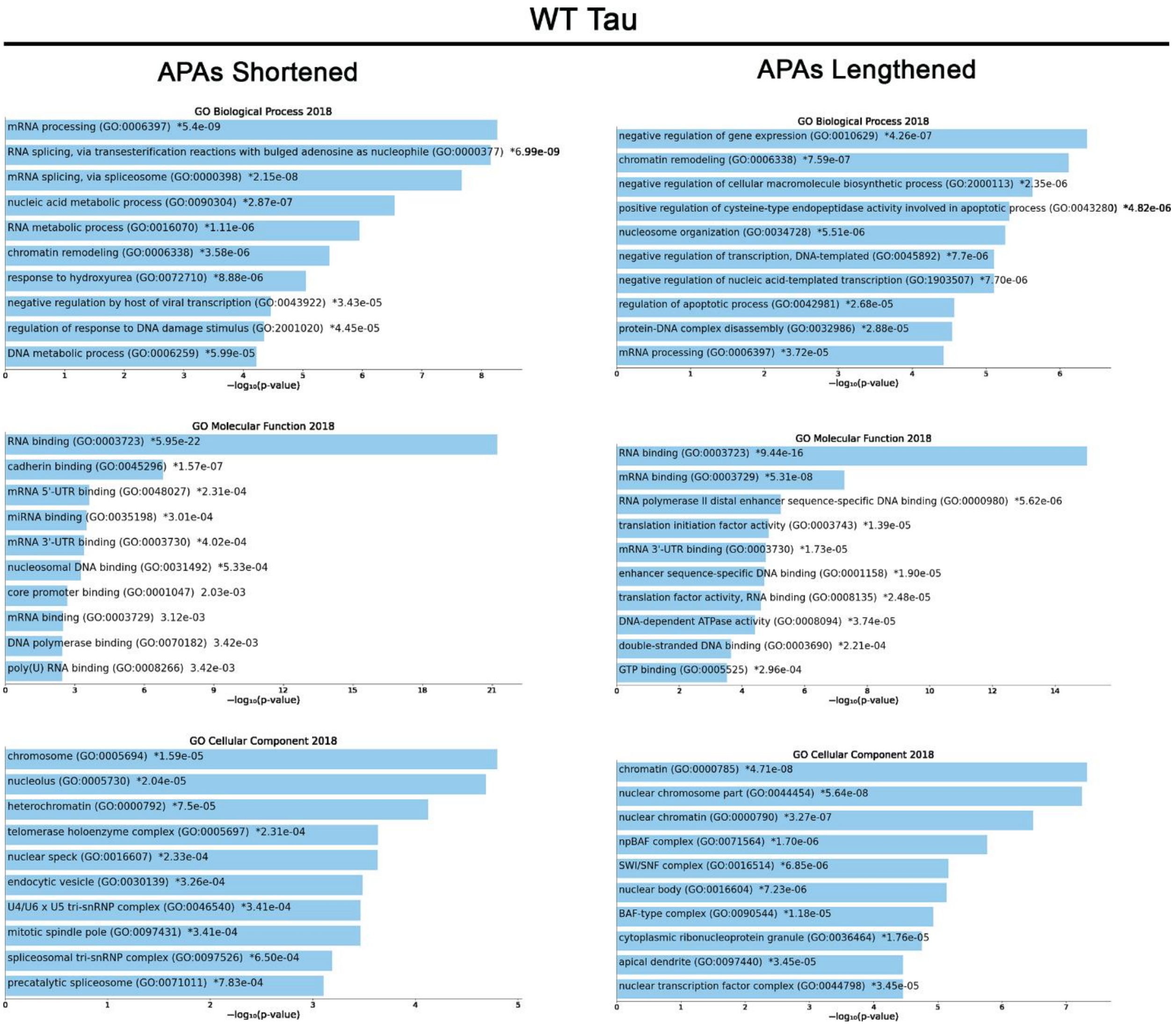

Fig S4. WT Tau 3'UTR shortened, and lengthened GO divided by Biological process, Molecular Function and Cellular Components.

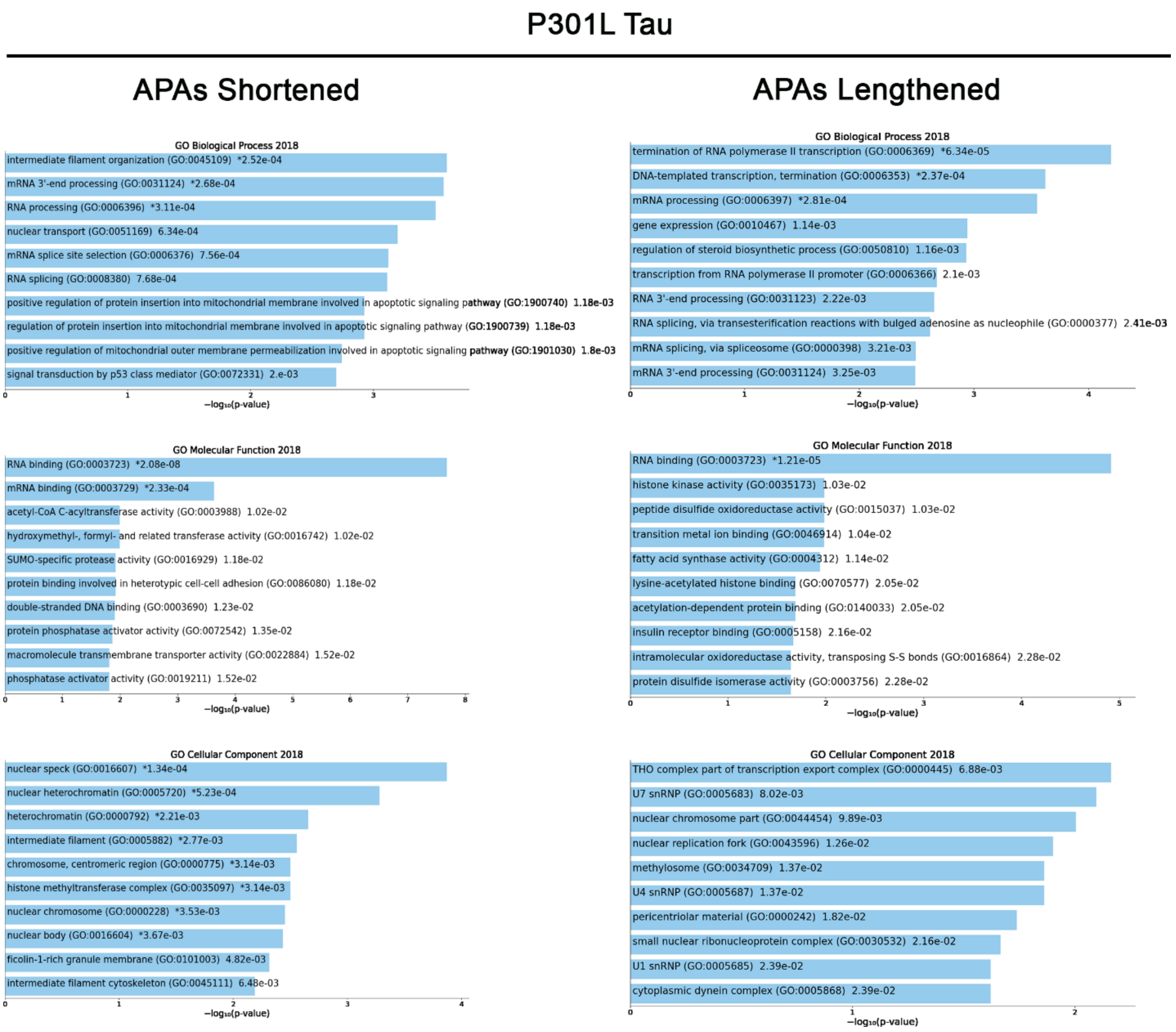

Fig S5. P301LTau 3'UTR shortened, and lengthened GO divided by Biological process, Molecular Function and Cellular Components.
