# Supplementary figures and images for: Tau Modulates mRNA Transcription, Alternative Polyadenylation Profiles of hnRNPs, Chromatin Remodeling and Spliceosome Complexes
Source: Front Mol Neurosci. 2021 Dec 3;14:742790. doi: 10.3389/fnmol.2021.742790 (PMC8678415; doi:10.3389/fnmol.2021.742790)

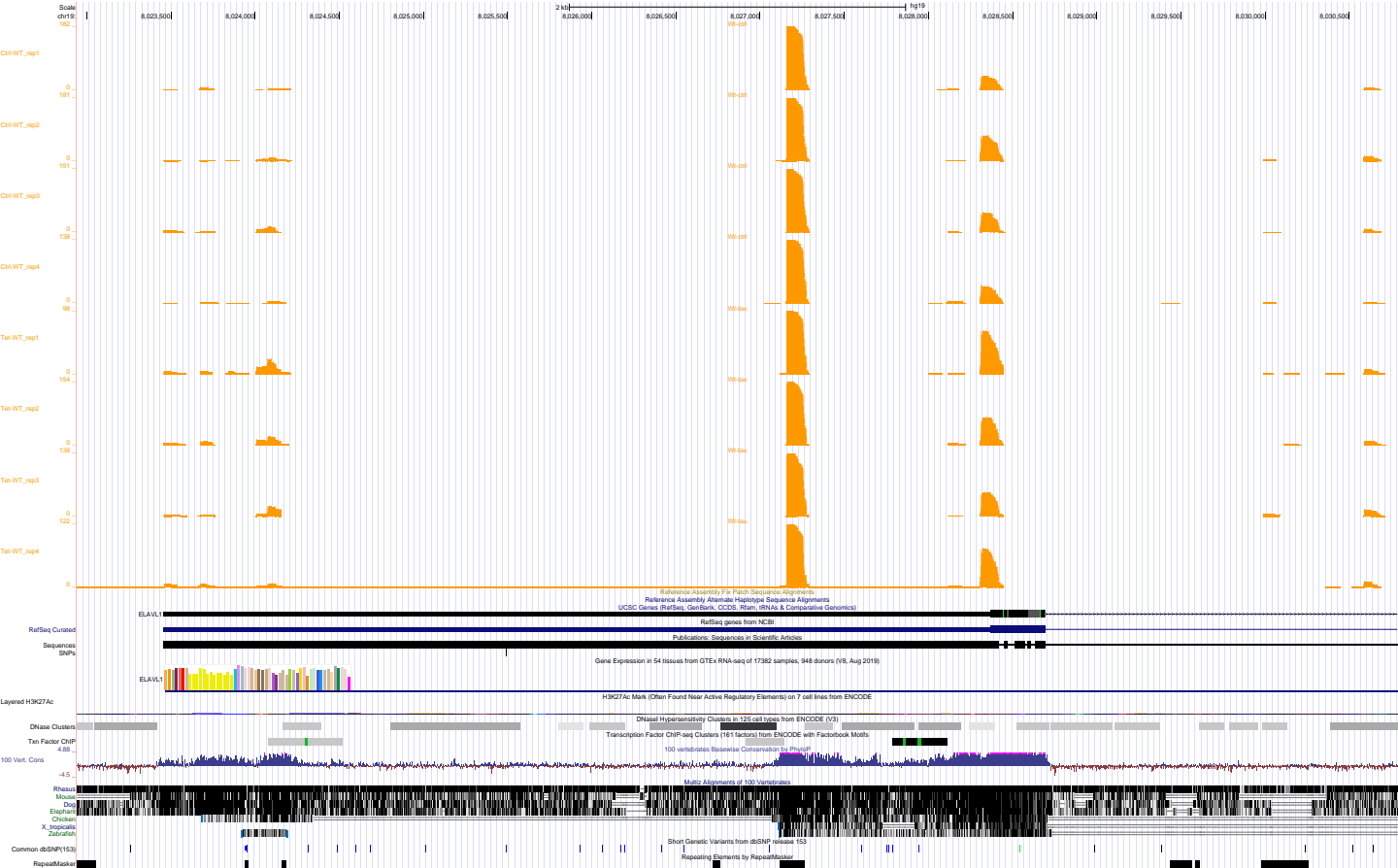

Supplement: Supplementary file 2 [file Data_Sheet_2.PDF]

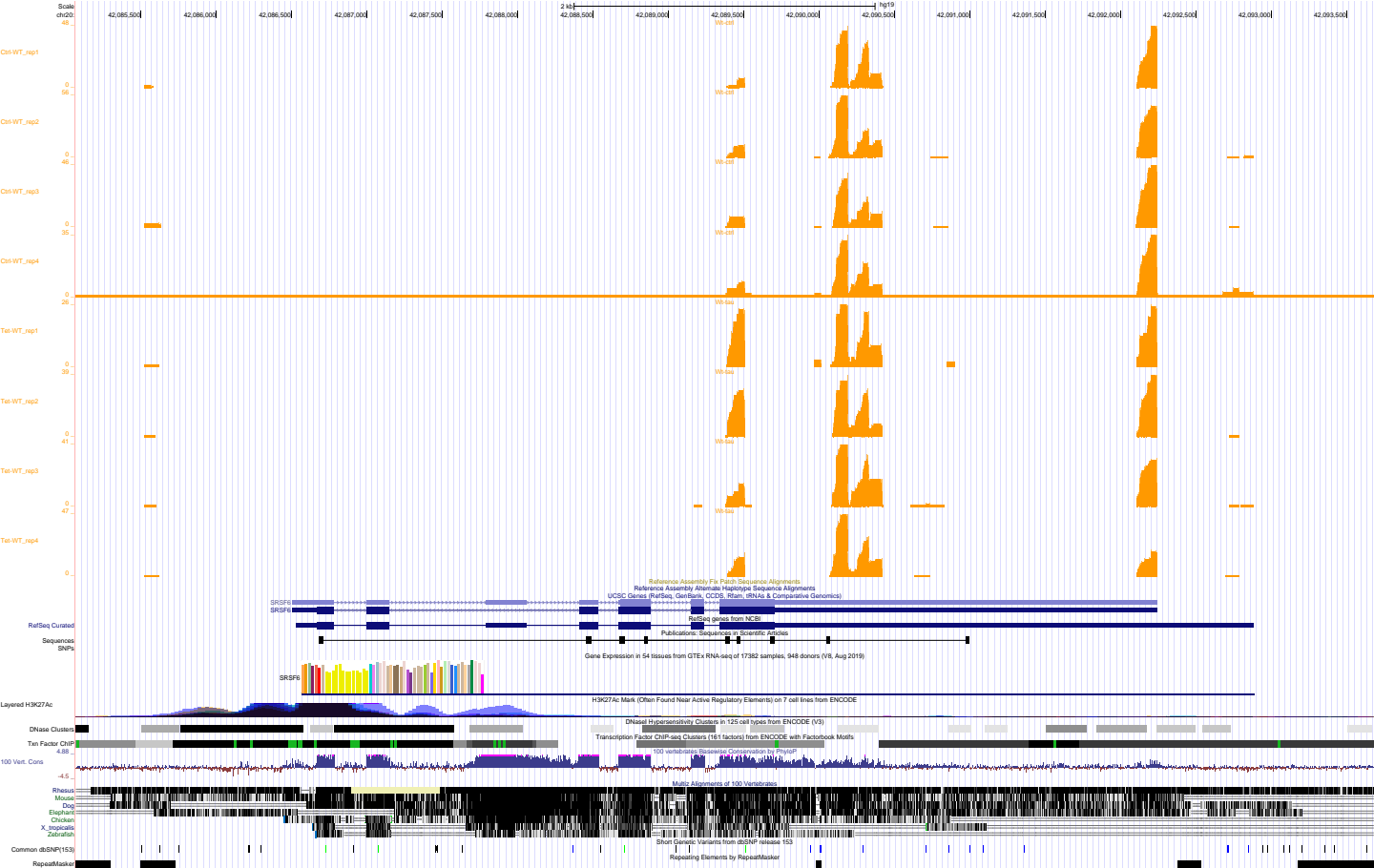

Supplement: Supplementary file 3 [file Data_Sheet_3.PDF]

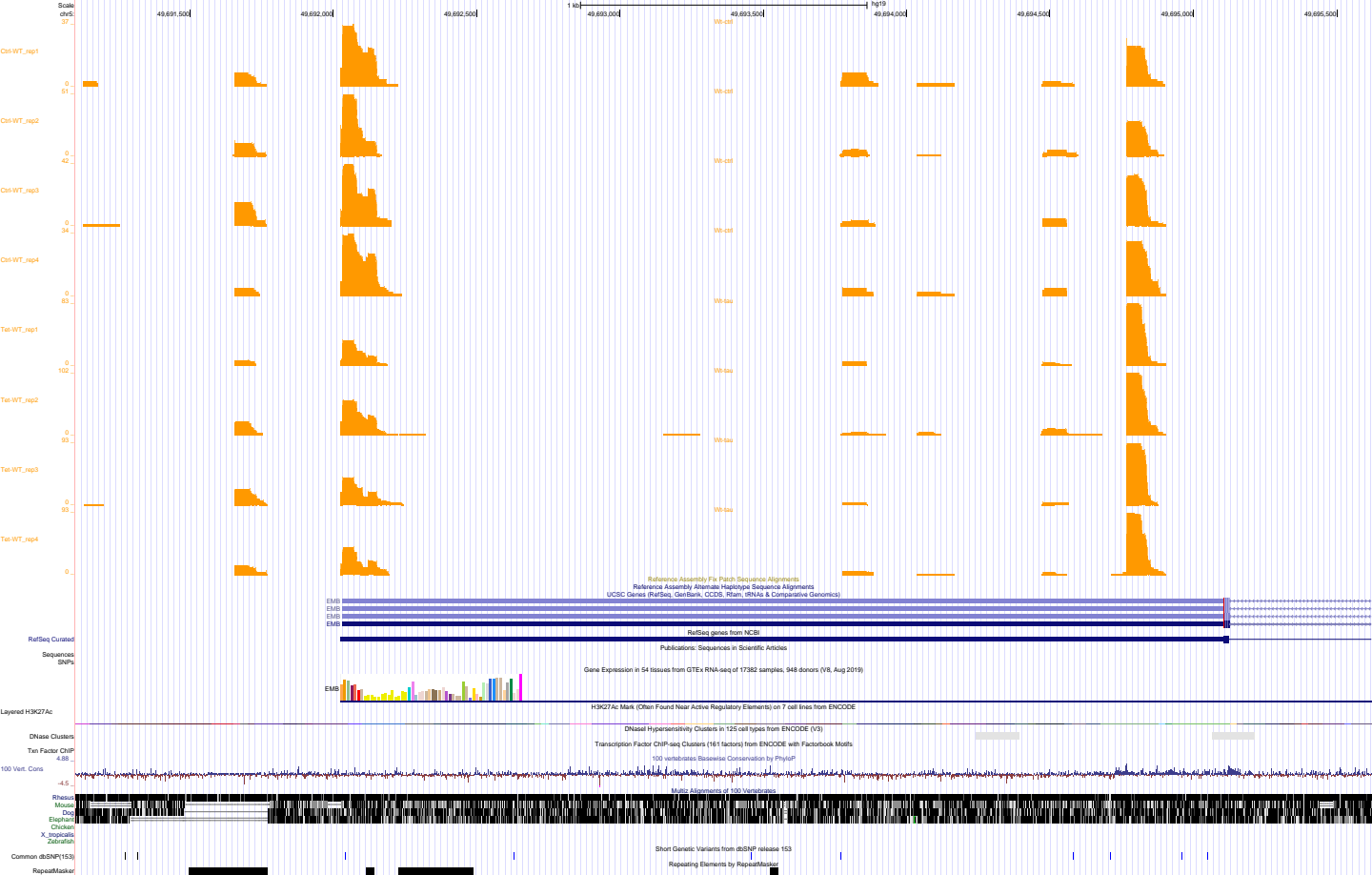

Supplement: Supplementary file 4 [file Data_Sheet_4.PDF]

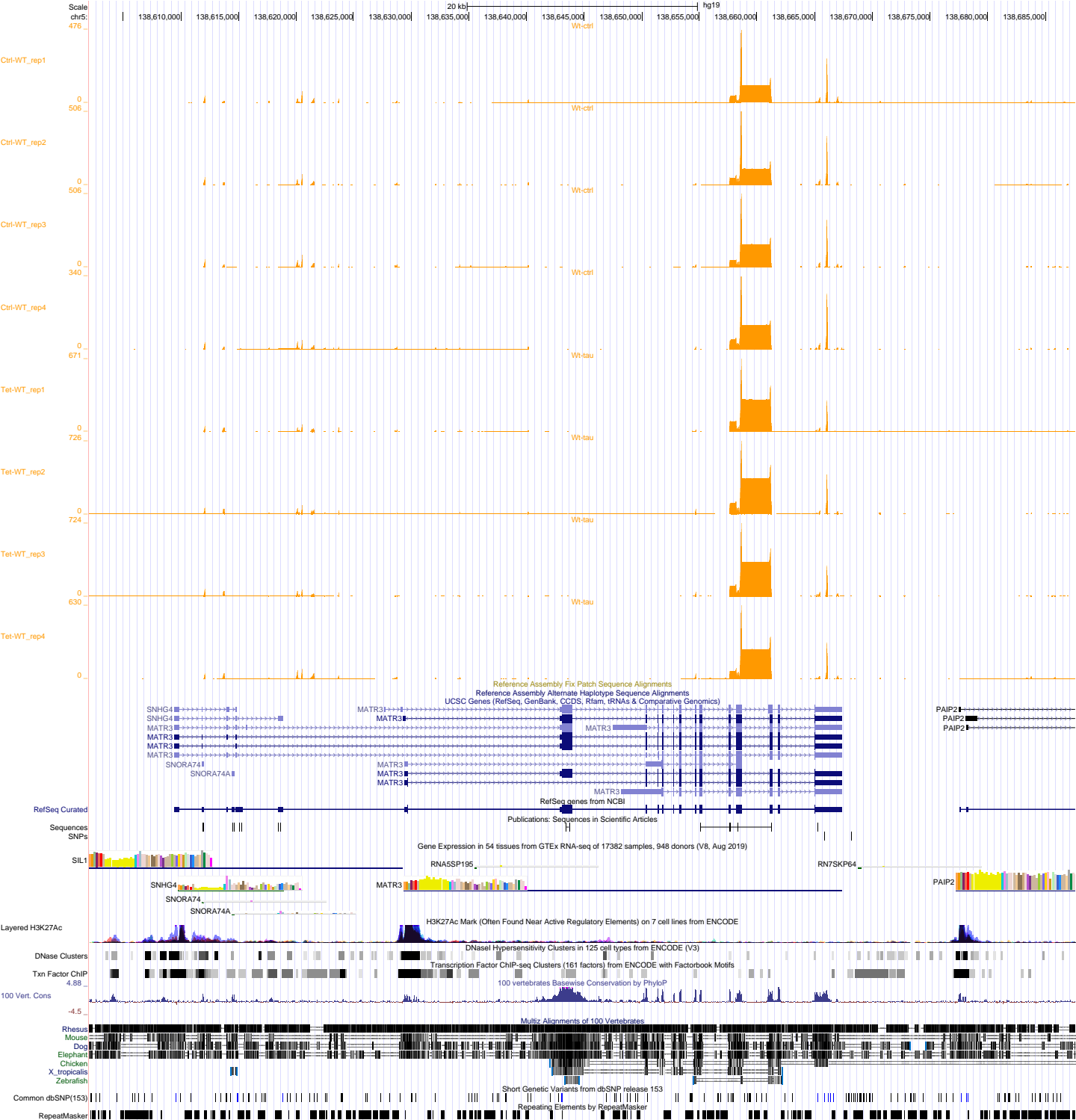

Supplement: Supplementary file 5 [file Data_Sheet_5.PDF]
