# Supplementary material for: Tau Modulates mRNA Transcription, Alternative Polyadenylation Profiles of hnRNPs, Chromatin Remodeling and Spliceosome Complexes
Source: Front Mol Neurosci. 2021 Dec 3;14:742790. doi: 10.3389/fnmol.2021.742790 (PMC8678415; doi:10.3389/fnmol.2021.742790)
Supplement: Supplementary file 6 [file Table_1.DOCX]

| Supplemental Table 1. Up-Regulated Genes by WT Tau | | | | | |
| --- | --- | --- | --- | --- | --- |
| Term | **P-value** | **Adjusted P-value** | **Odds Ratio** | **Combined Score** | **Genes** |
| cytoskeleton (GO:0005856) | 1.73E-05 | 0.007735674 | 4.807692308 | 52.70303651 | TUBA1B;SAP30BP;TUBB2B;TUBA1A;TMOD3;MAP7;TARS;TACC1;MAPT;CLU;RHOQ |
| polymeric cytoskeletal fiber (GO:0099513) | 4.34E-04 | 0.096694562 | 6.170300288 | 47.77891745 | TUBA1B;TUBB2B;TUBA1A;TUBB2A;MAPT;RHOQ |
| nuclear chromatin (GO:0000790) | 8.81E-04 | 0.130903368 | 5.38986705 | 37.91772839 | MEF2A;ZEB2;ANP32E;SUDS3;HIST2H2AC;HIST1H1C |
| microtubule cytoskeleton (GO:0015630) | 0.001616162 | 0.180202106 | 4.100281162 | 26.35538067 | TUBA1B;TUBB2B;TUBA1A;TUBB2A;MAP7;TACC1;MAPT |
| nuclear chromosome part (GO:0044454) | 0.001713127 | 0.152810938 | 4.058441558 | 25.84997914 | MEF2A;ZEB2;POLE3;ANP32E;SUDS3;CDC73;HIST2H2AC |
| microtubule (GO:0005874) | 0.00235399 | 0.17497991 | 5.411255411 | 32.74698914 | TUBA1B;TUBB2B;TUBA1A;TUBB2A;MAPT |
| preribosome, large subunit precursor (GO:0030687) | 0.00580801 | 0.370053218 | 17.48251748 | 90.00904291 | RRP15;FTSJ3 |
| nuclear speck (GO:0016607) | 0.009908573 | 0.552402927 | 3.839066339 | 17.71481484 | USP36;CARMIL1;GTF2H2C;BAZ2A;MAPT |
| cytoplasmic vesicle (GO:0031410) | 0.015007567 | 0.743708322 | 4.22832981 | 17.75560565 | TFRC;ANP32E;CLCN3;RHOQ |
| nuclear body (GO:0016604) | 0.019146203 | 0.853920672 | 2.574286555 | 10.18297877 | USP36;CARMIL1;GTF2H2C;BAZ2A;MAPT;SUDS3;SENP2 |
| axolemma (GO:0030673) | 0.026114353 | 1 | 37.87878788 | 138.0784165 | MAPT |
| nucleolus (GO:0005730) | 0.029395392 | 1 | 2.353415815 | 8.300303054 | USP36;NOM1;ZEB2;BAZ2A;PHF6;PELP1;FTSJ3 |
| messenger ribonucleoprotein complex (GO:1990124) | 0.030400721 | 1 | 32.46753247 | 113.4184725 | CPEB4 |
| spherical high-density lipoprotein particle (GO:0034366) | 0.034668438 | 1 | 28.40909091 | 95.50924912 | CLU |
| HFE-transferrin receptor complex (GO:1990712) | 0.034668438 | 1 | 28.40909091 | 95.50924912 | TFRC |
| Cdc73/Paf1 complex (GO:0016593) | 0.034668438 | 0.966382714 | 28.40909091 | 95.50924912 | CDC73 |
| apical dendrite (GO:0097440) | 0.034668438 | 0.909536672 | 28.40909091 | 95.50924912 | CLU |
| chromatin silencing complex (GO:0005677) | 0.034668438 | 0.859006857 | 28.40909091 | 95.50924912 | BAZ2A |
| Swr1 complex (GO:0000812) | 0.038917584 | 0.913539084 | 25.25252525 | 81.97750232 | ANP32E |
| chromatin (GO:0000785) | 0.041696789 | 0.929838401 | 3.071253071 | 9.758388048 | MEF2A;ZEB2;HIST2H2AC;PELP1 |
| preribosome (GO:0030684) | 0.043179537 | 0.917051121 | 6.060606061 | 19.04477924 | RRP15;FTSJ3 |
| proton-transporting V-type ATPase complex (GO:0033176) | 0.047360482 | 0.960126139 | 20.66115702 | 63.01584932 | ATP6V1D |
| ribonucleoprotein granule (GO:0035770) | 0.04848675 | 0.940221322 | 5.681818182 | 17.19582226 | TUBA1A;MAPT |
| holo TFIIH complex (GO:0005675) | 0.051554392 | 0.95805246 | 18.93939394 | 56.1575353 | GTF2H2C |
| Sin3 complex (GO:0016580) | 0.055730049 | 0.994224071 | 17.48251748 | 50.47615038 | SUDS3 |
| DNA-directed RNA polymerase II, holoenzyme (GO:0016591) | 0.059760267 | 1 | 5.050505051 | 14.22936503 | GTF2H2C;CDC73 |
| cytoplasmic exosome (RNase complex) (GO:0000177) | 0.064026913 | 1 | 15.15151515 | 41.64320855 | SUPV3L1 |
| keratin filament (GO:0045095) | 0.064026913 | 1 | 15.15151515 | 41.64320855 | TCHP |
| pericentriolar material (GO:0000242) | 0.068148277 | 1 | 14.20454545 | 38.15439487 | HOOK3 |
| nuclear replisome (GO:0043601) | 0.068148277 | 1 | 14.20454545 | 38.15439487 | POLE3 |
| Sin3-type complex (GO:0070822) | 0.068148277 | 0.980455861 | 14.20454545 | 38.15439487 | SUDS3 |
| dendrite (GO:0030425) | 0.06924185 | 0.965058279 | 3.171247357 | 8.46770561 | MAPT;CLU;CPEB4 |
| nuclear euchromatin (GO:0005719) | 0.076337257 | 1 | 12.62626263 | 32.48224959 | HIST1H1C |
| striated muscle thin filament (GO:0005865) | 0.080405026 | 1 | 11.96172249 | 30.15165779 | TMOD3 |
| euchromatin (GO:0000791) | 0.088487507 | 1 | 10.82251082 | 26.2434405 | HIST1H1C |
| transcriptionally active chromatin (GO:0035327) | 0.09250237 | 1 | 10.33057851 | 24.59215918 | PELP1 |
| histone acetyltransferase complex (GO:0000123) | 0.09250237 | 1 | 10.33057851 | 24.59215918 | KAT7 |
| INO80-type complex (GO:0097346) | 0.09250237 | 1 | 10.33057851 | 24.59215918 | ANP32E |
| recycling endosome (GO:0055037) | 0.09666912 | 1 | 3.819709702 | 8.924603778 | TUBA1A;TFRC |
| contractile fiber (GO:0043292) | 0.108387738 | 1 | 8.741258741 | 19.42342931 | TMOD3 |
| MLL1 complex (GO:0071339) | 0.112315932 | 1 | 8.417508418 | 18.40437335 | PELP1 |
| myofibril (GO:0030016) | 0.112315932 | 1 | 8.417508418 | 18.40437335 | TMOD3 |
| MLL1/2 complex (GO:0044665) | 0.112315932 | 1 | 8.417508418 | 18.40437335 | PELP1 |
| main axon (GO:0044304) | 0.135528342 | 1 | 6.887052342 | 13.76428715 | MAPT |
| actin cytoskeleton (GO:0015629) | 0.139839351 | 1 | 2.319109462 | 4.562293623 | TMOD3;TARS;RHOQ |
| centrosome (GO:0005813) | 0.145540984 | 1 | 1.971997634 | 3.800626224 | TCHP;TRAF3IP1;HOOK3;ATP6V1D |
| specific granule (GO:0042581) | 0.1565438 | 1 | 2.840909091 | 5.268237029 | CLCN3;ATP6V1D |
| contractile actin filament bundle (GO:0097517) | 0.161851895 | 1 | 5.681818182 | 10.34700903 | DAAM1 |
| integral component of Golgi membrane (GO:0030173) | 0.161851895 | 1 | 5.681818182 | 10.34700903 | UBIAD1 |
| stress fiber (GO:0001725) | 0.161851895 | 1 | 5.681818182 | 10.34700903 | DAAM1 |
| heterochromatin (GO:0000792) | 0.165547132 | 1 | 5.543237251 | 9.969508533 | BAZ2A |
| cytoplasmic ribonucleoprotein granule (GO:0036464) | 0.172060346 | 1 | 2.673796791 | 4.705641744 | TUBA1A;MAPT |
| intermediate filament (GO:0005882) | 0.183783091 | 1 | 4.940711462 | 8.369560635 | TCHP |
| microtubule organizing center (GO:0005815) | 0.184397492 | 1 | 1.793078716 | 3.031489277 | TCHP;TRAF3IP1;HOOK3;ATP6V1D |
| cis-Golgi network (GO:0005801) | 0.187382719 | 1 | 4.835589942 | 8.097689209 | HOOK3 |
| actomyosin (GO:0042641) | 0.190966652 | 1 | 4.734848485 | 7.839282495 | DAAM1 |
| actin filament (GO:0005884) | 0.215620364 | 1 | 4.132231405 | 6.339818153 | RHOQ |
| perinuclear region of cytoplasm (GO:0048471) | 0.232031019 | 1 | 1.803751804 | 2.635072538 | GALNT6;TFRC;CLU |
| PML body (GO:0016605) | 0.239531081 | 1 | 3.66568915 | 5.238534082 | SENP2 |
| platelet alpha granule lumen (GO:0031093) | 0.256167561 | 1 | 3.392130258 | 4.619821964 | CLU |
| nucleoplasm part (GO:0044451) | 0.266189763 | 1 | 1.675228948 | 2.217242288 | GTF2H2C;KAT7;SUDS3 |
| intermediate filament cytoskeleton (GO:0045111) | 0.269217242 | 1 | 3.201024328 | 4.200501392 | SAP30BP |
| lytic vacuole membrane (GO:0098852) | 0.273570882 | 1 | 1.950838861 | 2.528666646 | DAGLB;ATP6V1D |
| nuclear periphery (GO:0034399) | 0.291511782 | 1 | 2.913752914 | 3.591709949 | MAPT |
| clathrin-coated vesicle membrane (GO:0030665) | 0.300859484 | 1 | 2.805836139 | 3.370123323 | TFRC |
| phagocytic vesicle (GO:0045335) | 0.307023439 | 1 | 2.73822563 | 3.23338222 | CLCN3 |
| platelet alpha granule (GO:0031091) | 0.328177166 | 1 | 2.525252525 | 2.813640594 | CLU |
| specific granule membrane (GO:0035579) | 0.331146544 | 1 | 2.497502498 | 2.76022545 | ATP6V1D |
| clathrin-coated vesicle (GO:0030136) | 0.357293708 | 1 | 2.272727273 | 2.33908437 | TFRC |
| lysosomal membrane (GO:0005765) | 0.367155241 | 1 | 1.562011871 | 1.565089849 | DAGLB;ATP6V1D |
| nuclear chromosome, telomeric region (GO:0000784) | 0.376929429 | 1 | 2.12404418 | 2.072424172 | CDC73 |
| endocytic vesicle (GO:0030139) | 0.379685681 | 1 | 2.104377104 | 2.037903041 | CLCN3 |
| chromosome, telomeric region (GO:0000781) | 0.422181171 | 1 | 1.832844575 | 1.580499895 | CDC73 |
| microtubule organizing center part (GO:0044450) | 0.427286491 | 1 | 1.803751804 | 1.533731156 | HOOK3 |
| late endosome (GO:0005770) | 0.461796269 | 1 | 1.623376623 | 1.254271854 | CLCN3 |
| axon (GO:0030424) | 0.464181054 | 1 | 1.611863314 | 1.237073825 | MAPT |
| RNA polymerase II transcription factor complex (GO:0090575) | 0.478271942 | 1 | 1.546072975 | 1.140345997 | GTF2H2C |
| lysosome (GO:0005764) | 0.557038143 | 1 | 1.07712193 | 0.630247266 | DAGLB;ATP6V1D |
| Golgi membrane (GO:0000139) | 0.582354922 | 1 | 1.028383381 | 0.556021375 | GALNT6;DSE |
| early endosome (GO:0005769) | 0.626341943 | 1 | 1.023751024 | 0.478970947 | CLCN3 |
| Golgi subcompartment (GO:0098791) | 0.626391117 | 1 | 0.948946669 | 0.443898572 | GALNT6;DSE |
| mitochondrion (GO:0005739) | 0.667382777 | 1 | 0.886053518 | 0.358312528 | TCHP;SUPV3L1;MAPT;CLU |
| mitochondrial matrix (GO:0005759) | 0.745573163 | 1 | 0.737898465 | 0.216648473 | SUPV3L1 |
| secretory granule lumen (GO:0034774) | 0.755626978 | 1 | 0.716948666 | 0.20089435 | CLU |
| integral component of plasma membrane (GO:0005887) | 0.96056947 | 1 | 0.466041136 | 0.018748356 | TFRC;NRG3;CLCN3 |
