# Supplementary material for: Tau Modulates mRNA Transcription, Alternative Polyadenylation Profiles of hnRNPs, Chromatin Remodeling and Spliceosome Complexes
Source: Front Mol Neurosci. 2021 Dec 3;14:742790. doi: 10.3389/fnmol.2021.742790 (PMC8678415; doi:10.3389/fnmol.2021.742790)
Supplement: Supplementary file 7 [file Table_2.DOCX]

| Supplemental Table 2 - Down-Regulated Genes by WT Tau | | | | | |
| --- | --- | --- | --- | --- | --- |
| Term | **P-value** | **Adjusted P-value** | **Odds Ratio** | **Combined Score** | **Genes** |
| axon initial segment (GO:0043194) | 0.013421829 | 1 | 74.07407407 | 319.3239135 | KCNQ2 |
| node of Ranvier (GO:0033268) | 0.017856968 | 1 | 55.55555556 | 223.6311935 | KCNQ2 |
| main axon (GO:0044304) | 0.048367756 | 1 | 20.2020202 | 61.19034096 | KCNQ2 |
| SCF ubiquitin ligase complex (GO:0019005) | 0.077961385 | 1 | 12.34567901 | 31.50051407 | KLHL11 |
| coated vesicle (GO:0030135) | 0.101264667 | 1 | 9.389671362 | 21.5025138 | STX6 |
| ribosome (GO:0005840) | 0.108009525 | 1 | 8.771929825 | 19.52224439 | NCK1 |
| perinuclear region of cytoplasm (GO:0048471) | 0.109697823 | 1 | 3.527336861 | 7.795505317 | STC2;STX6 |
| trans-Golgi network membrane (GO:0032588) | 0.120027161 | 1 | 7.843137255 | 16.62774289 | STX6 |
| clathrin-coated vesicle (GO:0030136) | 0.139709564 | 1 | 6.666666667 | 13.1212637 | STX6 |
| Golgi membrane (GO:0000139) | 0.141642689 | 1 | 3.016591252 | 5.895769726 | STX6;BLZF1 |
| Golgi subcompartment (GO:0098791) | 0.160952725 | 1 | 2.783576896 | 5.084605682 | STX6;BLZF1 |
| mitochondrion (GO:0005739) | 0.197703841 | 1 | 1.949317739 | 3.159815052 | TRUB1;OXCT1;PFDN2 |
| cullin-RING ubiquitin ligase complex (GO:0031461) | 0.238857152 | 1 | 3.683241252 | 5.273994836 | KLHL11 |
| trans-Golgi network (GO:0005802) | 0.243455603 | 1 | 3.603603604 | 5.091245694 | STX6 |
| nucleolus (GO:0005730) | 0.269438542 | 1 | 1.972386588 | 2.586617276 | UBE2T;UPF3A |
| early endosome (GO:0005769) | 0.284737465 | 1 | 3.003003003 | 3.772335434 | STX6 |
| endoplasmic reticulum lumen (GO:0005788) | 0.335058182 | 1 | 2.469135802 | 2.699879222 | STC2 |
| mitochondrial matrix (GO:0005759) | 0.37245128 | 1 | 2.164502165 | 2.13776849 | OXCT1 |
| integral component of plasma membrane (GO:0005887) | 0.377736622 | 1 | 1.367053999 | 1.330906484 | SLC26A2;KCNQ2;ICAM5 |
