# Supplementary material for: Tau Modulates mRNA Transcription, Alternative Polyadenylation Profiles of hnRNPs, Chromatin Remodeling and Spliceosome Complexes
Source: Front Mol Neurosci. 2021 Dec 3;14:742790. doi: 10.3389/fnmol.2021.742790 (PMC8678415; doi:10.3389/fnmol.2021.742790)
Supplement: Supplementary file 8 [file Table_3.DOCX]

| Supplemental Table 3. Up-Regulated Genes by P301L Tau | | | | | |
| --- | --- | --- | --- | --- | --- |
| Term | **P-value** | **Adjusted P-value** | **Odds Ratio** | **Combined Score** | **Genes** |
| axolemma (GO:0030673) | 0.002996578 | 1 | 333.3333333 | 1936.761417 | MAPT |
| dendrite (GO:0030425) | 0.004890312 | 1 | 18.60465116 | 98.98603085 | NLGN1;MAPT |
| filopodium tip (GO:0032433) | 0.004989818 | 0.741819593 | 200 | 1060.071173 | NLGN1 |
| spanning component of membrane (GO:0089717) | 0.005487568 | 0.611863795 | 181.8181818 | 946.412758 | NLGN1 |
| nuclear speck (GO:0016607) | 0.009082553 | 0.810163748 | 13.51351351 | 63.53243152 | ITPKC;MAPT |
| microtubule cytoskeleton (GO:0015630) | 0.015239427 | 1 | 10.30927835 | 43.13267377 | FER;MAPT |
| main axon (GO:0044304) | 0.016381548 | 1 | 60.60606061 | 249.1878617 | MAPT |
| cytoskeleton (GO:0005856) | 0.026441482 | 1 | 7.692307692 | 27.94477859 | FER;MAPT |
| filopodium (GO:0030175) | 0.029604609 | 1 | 33.33333333 | 117.3275068 | NLGN1 |
| nuclear body (GO:0016604) | 0.036393121 | 1 | 6.472491909 | 21.44579618 | ITPKC;MAPT |
| nuclear periphery (GO:0034399) | 0.038330884 | 1 | 25.64102564 | 83.62818815 | MAPT |
| ribonucleoprotein granule (GO:0035770) | 0.039296097 | 1 | 25 | 80.91575164 | MAPT |
| axon (GO:0030424) | 0.068319537 | 1 | 14.18439716 | 38.0646738 | MAPT |
| RNA polymerase II transcription factor complex (GO:0090575) | 0.07113123 | 1 | 13.60544218 | 35.9622965 | FOS |
| cytoplasmic ribonucleoprotein granule (GO:0036464) | 0.081838782 | 1 | 11.76470588 | 29.44710636 | MAPT |
| microtubule (GO:0005874) | 0.100196316 | 1 | 9.523809524 | 21.91070345 | MAPT |
| polymeric cytoskeletal fiber (GO:0099513) | 0.105186383 | 1 | 9.049773756 | 20.38028436 | MAPT |
| nuclear chromatin (GO:0000790) | 0.119561652 | 1 | 7.90513834 | 16.78990615 | FER |
| actin cytoskeleton (GO:0015629) | 0.137676093 | 1 | 6.802721088 | 13.48878577 | FER |
| chromatin (GO:0000785) | 0.138551081 | 1 | 6.756756757 | 13.35483925 | FER |
| nuclear chromosome part (GO:0044454) | 0.179622583 | 1 | 5.102040816 | 8.759680555 | FER |
| nucleolus (GO:0005730) | 0.291015997 | 1 | 2.958579882 | 3.652003078 | TSPYL2 |
| mitochondrion (GO:0005739) | 0.409477333 | 1 | 1.949317739 | 1.740494599 | MAPT |
| integral component of plasma membrane (GO:0005887) | 0.532245452 | 1 | 1.367053999 | 0.862133316 | NLGN1 |
