# Supplementary material for: Tau Modulates mRNA Transcription, Alternative Polyadenylation Profiles of hnRNPs, Chromatin Remodeling and Spliceosome Complexes
Source: Front Mol Neurosci. 2021 Dec 3;14:742790. doi: 10.3389/fnmol.2021.742790 (PMC8678415; doi:10.3389/fnmol.2021.742790)
Supplement: Supplementary file 9 [file Table_4.docx]

| Supplementary Table 4. Down-Regulated Genes by P301L Tau | | | | | |  |
| --- | --- | --- | --- | --- | --- | --- |
| Term | **P-value** | **Adjusted P-value** | **Odds Ratio** | **Combined Score** | **Genes** | |
| Cul4-RING E3 ubiquitin ligase complex (GO:0080008) | 0.0018 | 0.80278471 | 555.5556 | 3511.104 | DCAF12 | |
| cullin-RING ubiquitin ligase complex (GO:0031461) | 0.00905 | 1 | 110.4972 | 519.8896 | DCAF12 | |
| centrosome (GO:0005813) | 0.02305 | 1 | 43.38395 | 163.5617 | DCAF12 | |
| microtubule organizing center (GO:0005815) | 0.02535 | 1 | 39.44773 | 144.9697 | DCAF12 | |
